# Supplementary figures and images for: Ubp2 modulates DJ-1-mediated redox-dependent mitochondrial dynamics in Saccharomyces cerevisiae
Source: PLoS Genet. 2025 Jul 3;21(7):e1011353. doi: 10.1371/journal.pgen.1011353 (PMC12251144; doi:10.1371/journal.pgen.1011353)

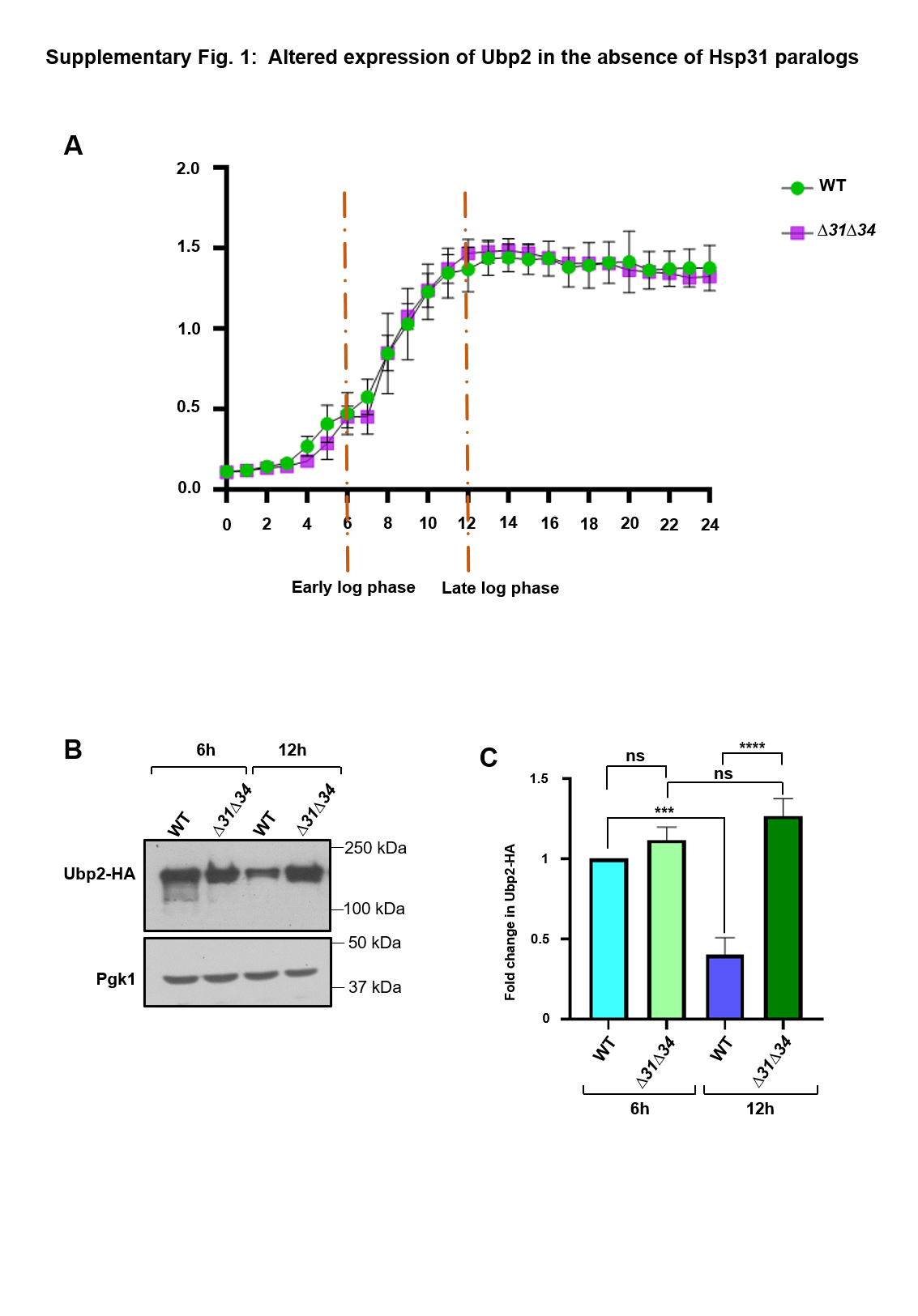

Supplement: S1 Fig — (A) WT and ∆31∆34 strains containing HA-tagged Ubp2 at the C-terminus were grown in dextrose-containing media for indicated time intervals. The early-log phase (6h) and late-log phase (12h) are highlighted. (B) The cell lysates prepared from strains at indicated time intervals were subjected to Ubp2 expression analysis by Western blotting. (C) The blots were quantified by densitometry, and the change in the expression levels of Ubp2-HA was represented. One-way ANOVA with Tukey’s multiple comparison test was used for significance analysis. Error bars represent the standard deviation in median values from 3 biological replicates. Asterisks indicate the p-value, *, p < 0.05; **, p < 0.01; ***, p < 0.001; ****, p < 0.0001. (TIFF) [file pgen.1011353.s001.tiff]

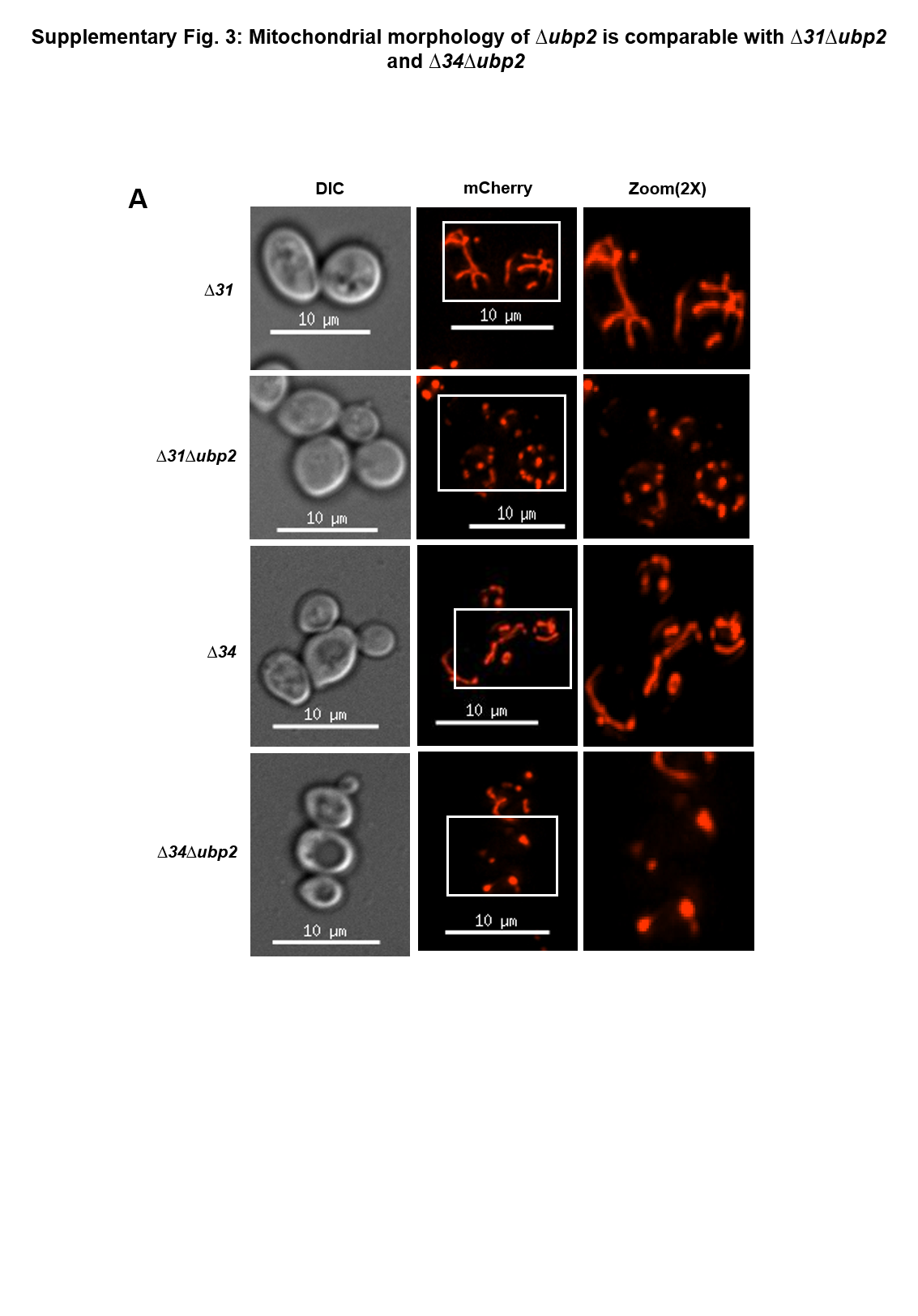

Supplement: S3 Fig — Mitochondrial morphology in the indicated strains transformed with mTS-mCherry was analyzed by microscopy. Scale bar (10 μm). (TIFF) [file pgen.1011353.s003.tiff]

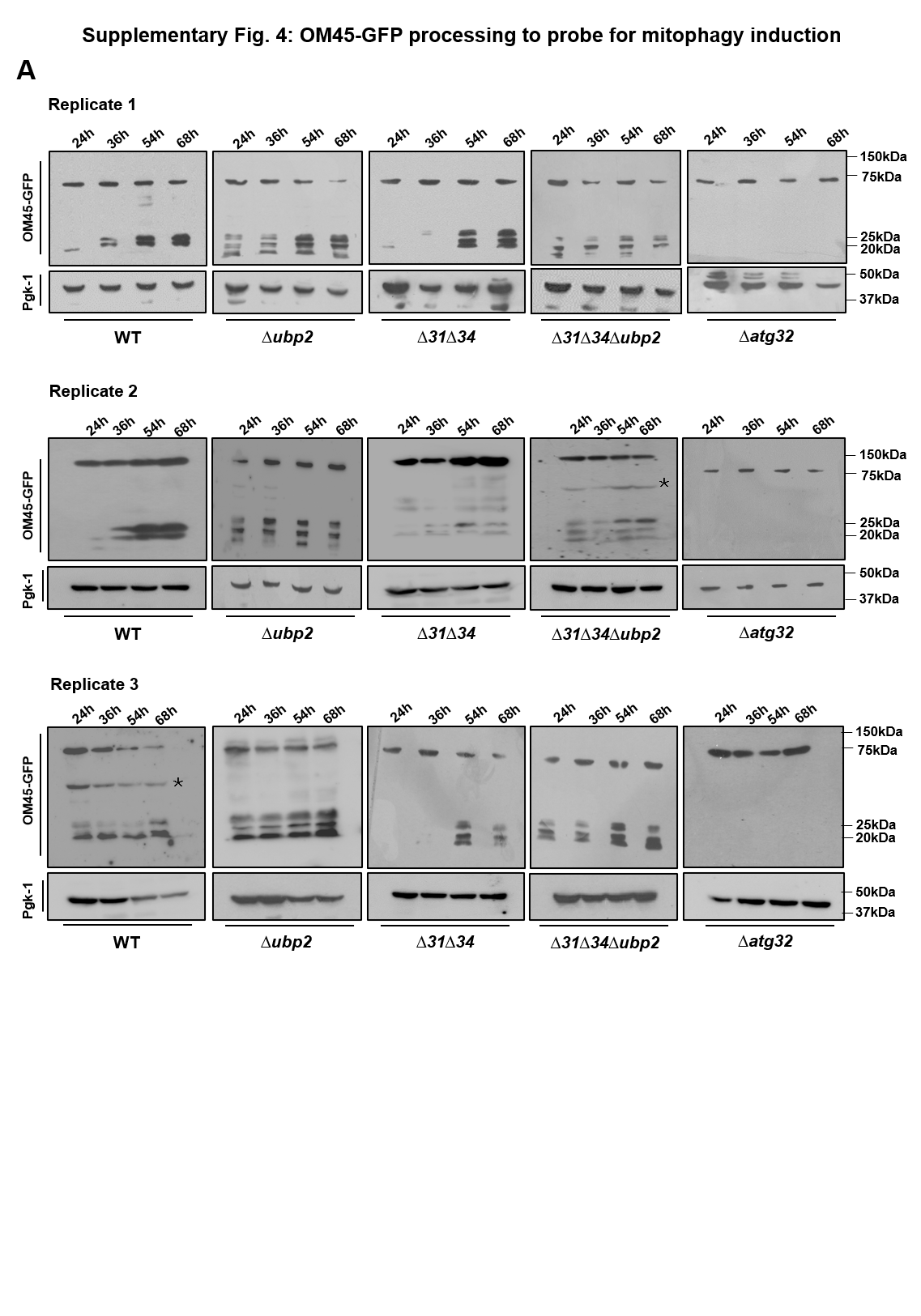

Supplement: S4 Fig — (A, B) The indicated strains were subjected to mitophagy induction, and lysates were collected at different time points. Western blotting was performed to probe the processed GFP (A) and quantified from the triplicates by densitometry. Graph depicting the ratio of free GFP by the total GFP (processed and unprocessed) quantified (B). (TIFF) [file pgen.1011353.s004.tiff]

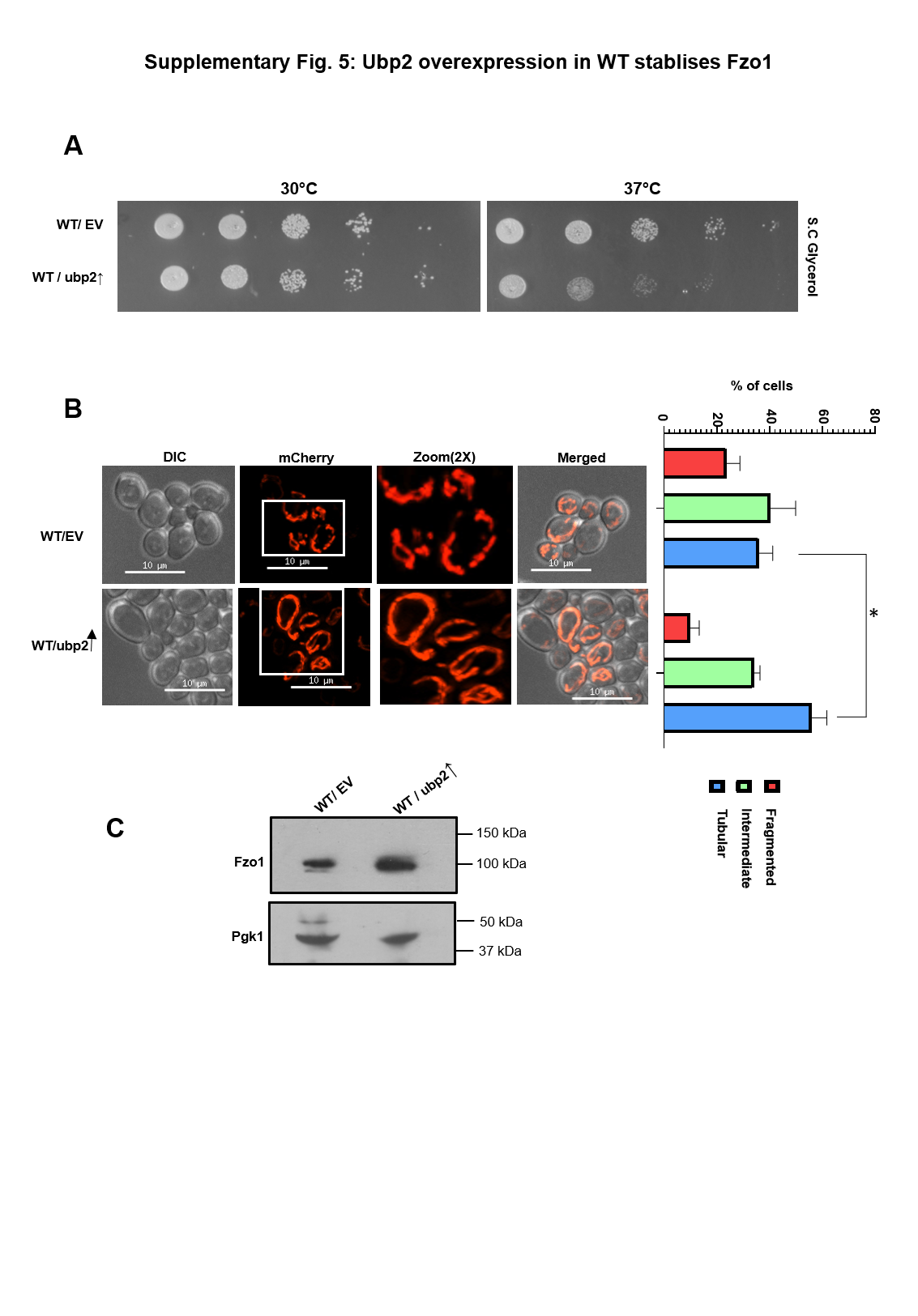

Supplement: S5 Fig — (A) Growth phenotype assessment. The indicated yeast strains were allowed to grow up to the mid-log phase in SC dextrose broth at 30°C. Ten-fold serially diluted cells were spotted on the indicated media and incubated at permissive temperature (30°C) and non-permissive temperature (37°C). Images were captured at 72h for glycerol. (B) Assessment of mitochondrial integrity. Yeast strains expressing MTS-mCherry grown in SC Leu- dextrose till the mid-log phase were subjected to microscopic analysis to visualize mitochondria. Scale bar (10 μm). (C) Lysates prepared from the cells were analyzed for the differences in Fzo1 expression by Western blotting. (TIFF) [file pgen.1011353.s005.tiff]

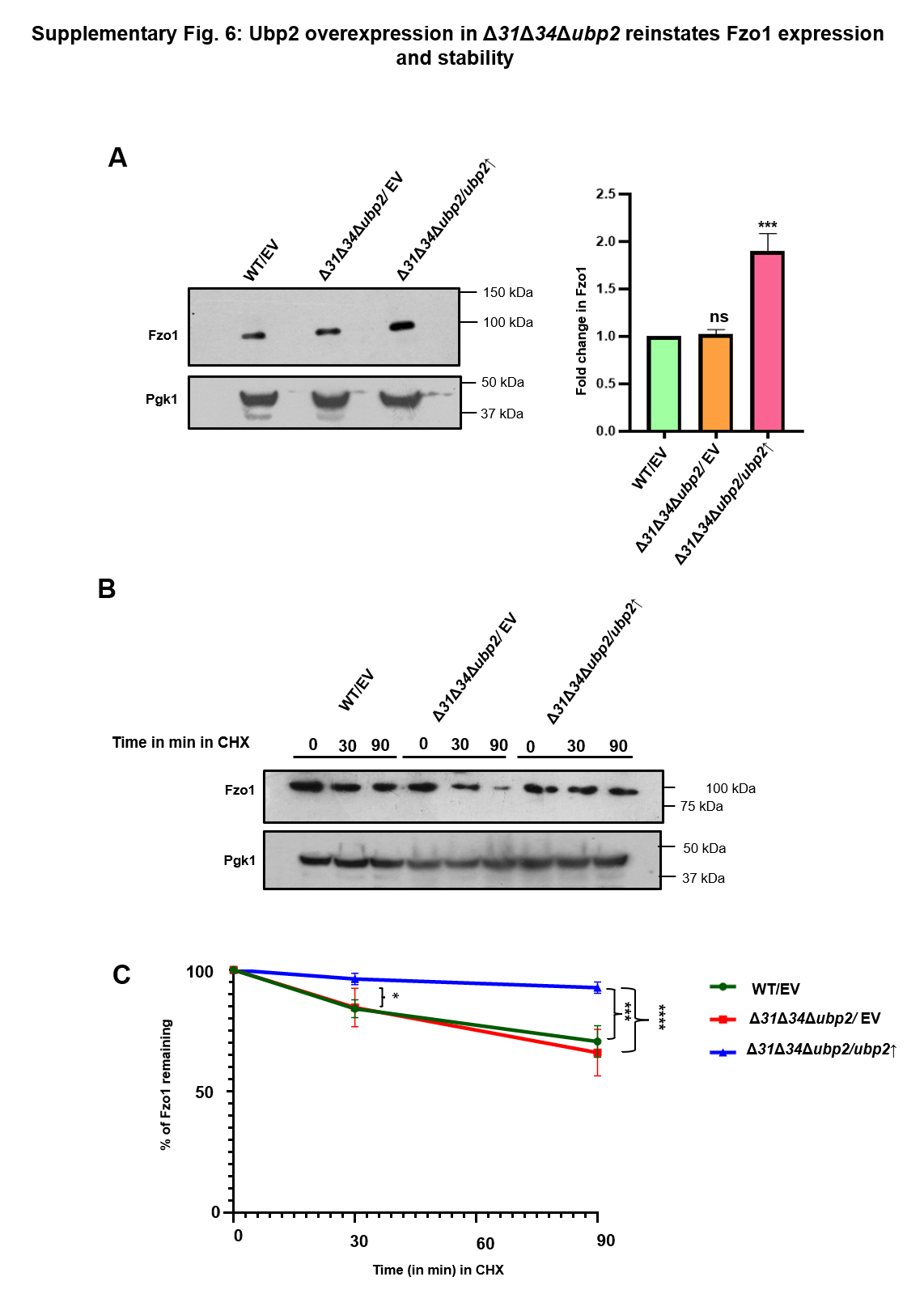

Supplement: S6 Fig — (A) Fzo1 steady-state levels were probed in the indicated strains by Western blotting using anti-Fzo1 antibody. (B) Cycloheximide Chase assay. The cells from yeast strains were subjected to treatment with cycloheximide for the indicated time points, and the lysates were subjected to western blotting using anti-Fzo1 antibody. (TIF) [file pgen.1011353.s006.tif]

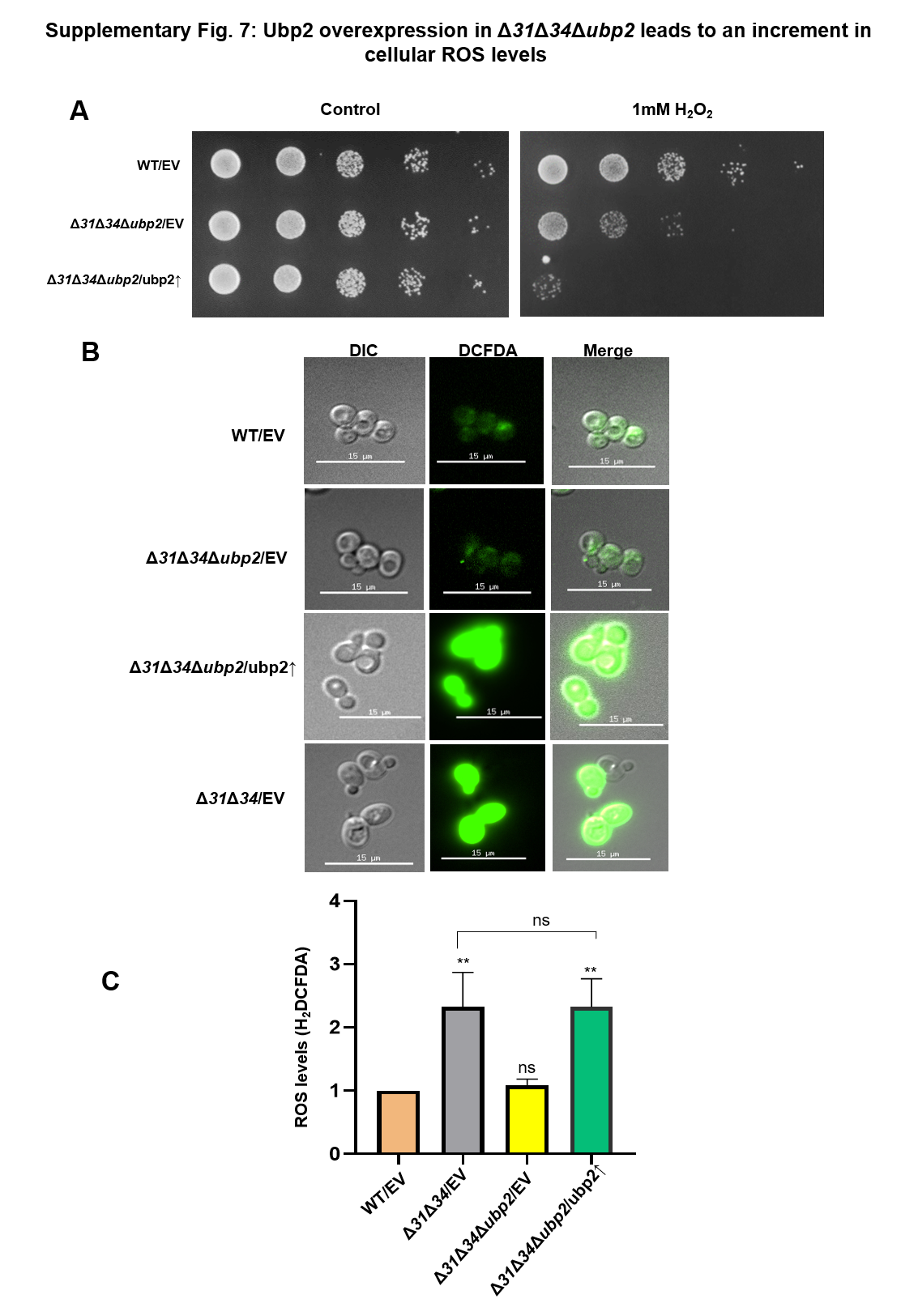

Supplement: S7 Fig — (A) Phenotypic analysis upon treatment with H2O2. Cells were treated with 1 mM H2O2 and spotted in SC-Leu-Dextrose plates. Images were captured after 36 h. (B) H2DCFDA treatment was performed to analyse ROS levels by microscopy. Scale bar (15 μm). (TIFF) [file pgen.1011353.s007.tiff]

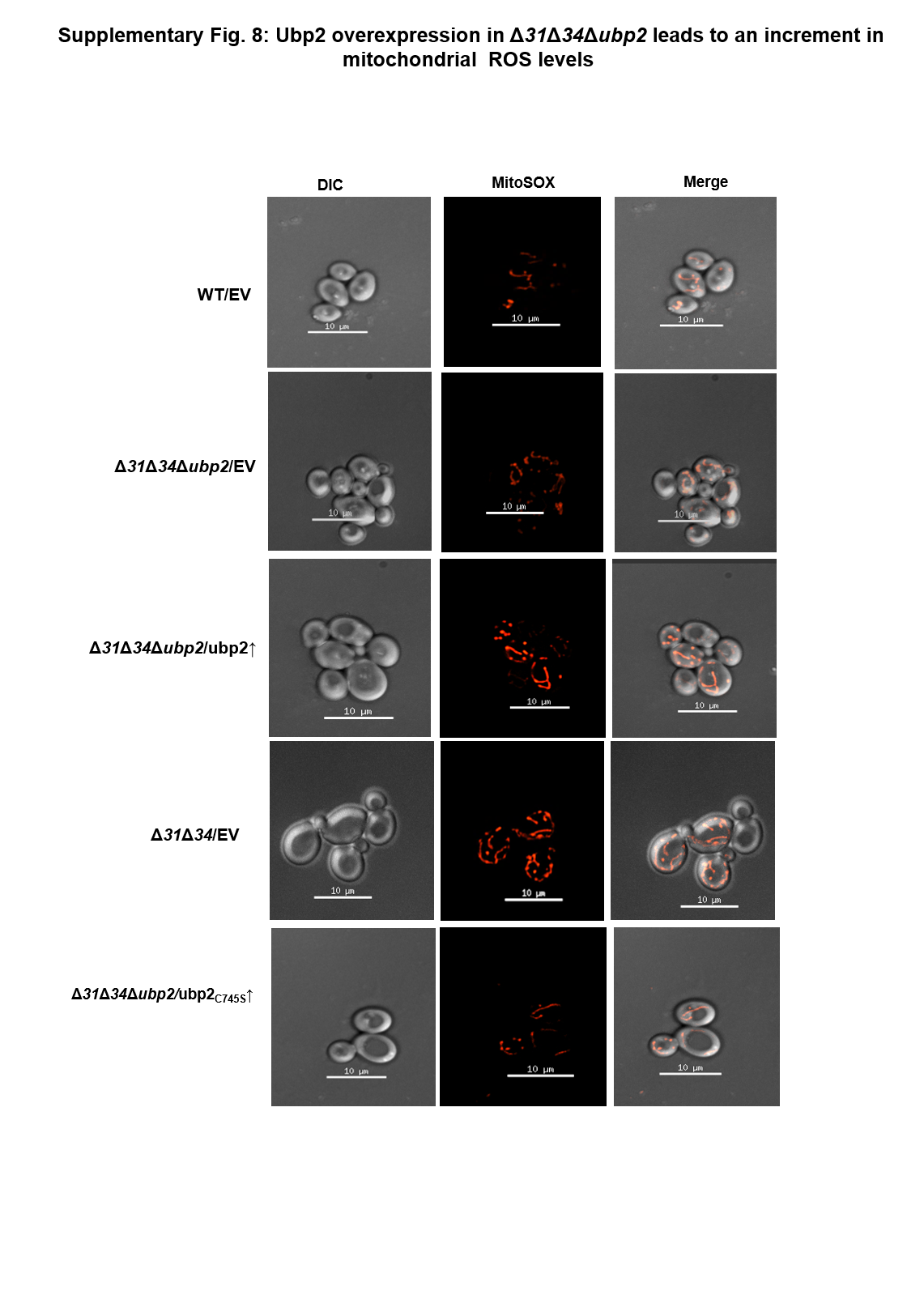

Supplement: S8 Fig — Cells were subjected to staining with MitoSOX to estimate mitochondrial basal ROS upon overexpression of Ubp2 and its cysteine mutant (Ubp2C745S). Scale bar (10 μm). (TIFF) [file pgen.1011353.s008.tiff]

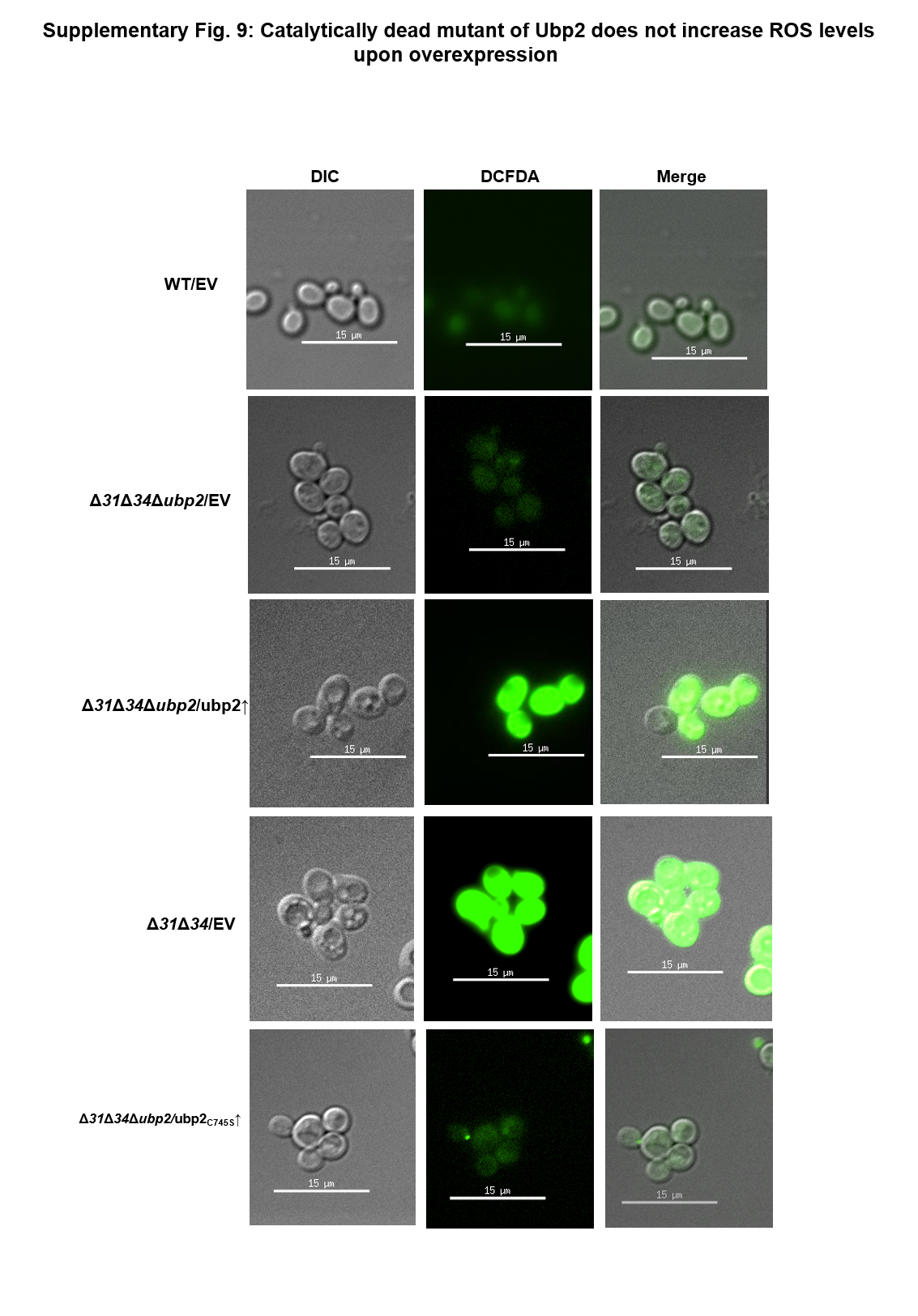

Supplement: S9 Fig — Overexpression of Ubp2 increases basal ROS levels,while the Ubp2C745S mutant does not affect the redox status.The indicated strains were stained with H2DCFDA and visualised by microscopy. Scale bar (15 μm). (TIFF) [file pgen.1011353.s009.tiff]

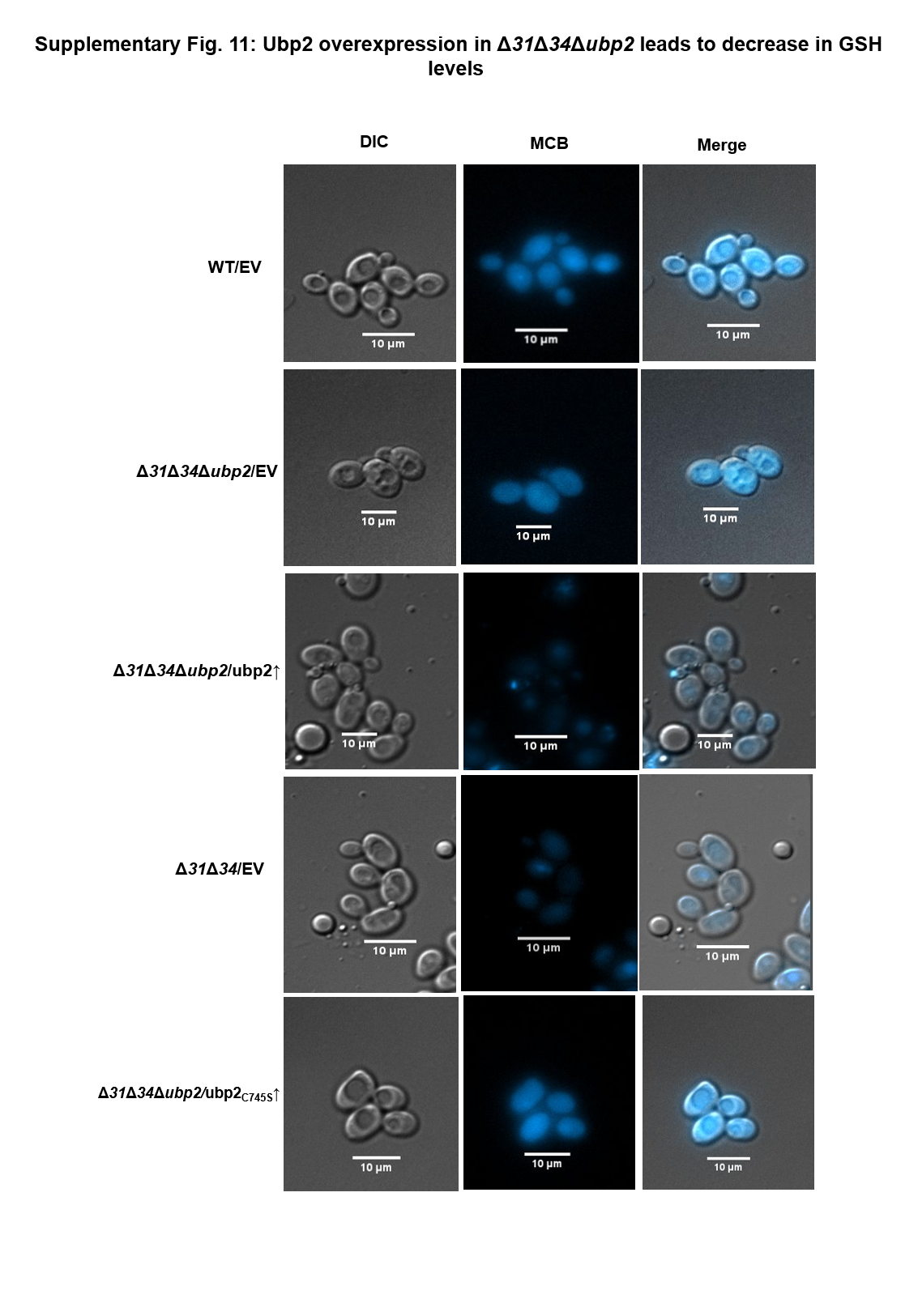

Supplement: S11 Fig — The indicated strains were stained with MCB and subjected to microscopy.Scale bar (10 μm). (TIFF) [file pgen.1011353.s011.tiff]

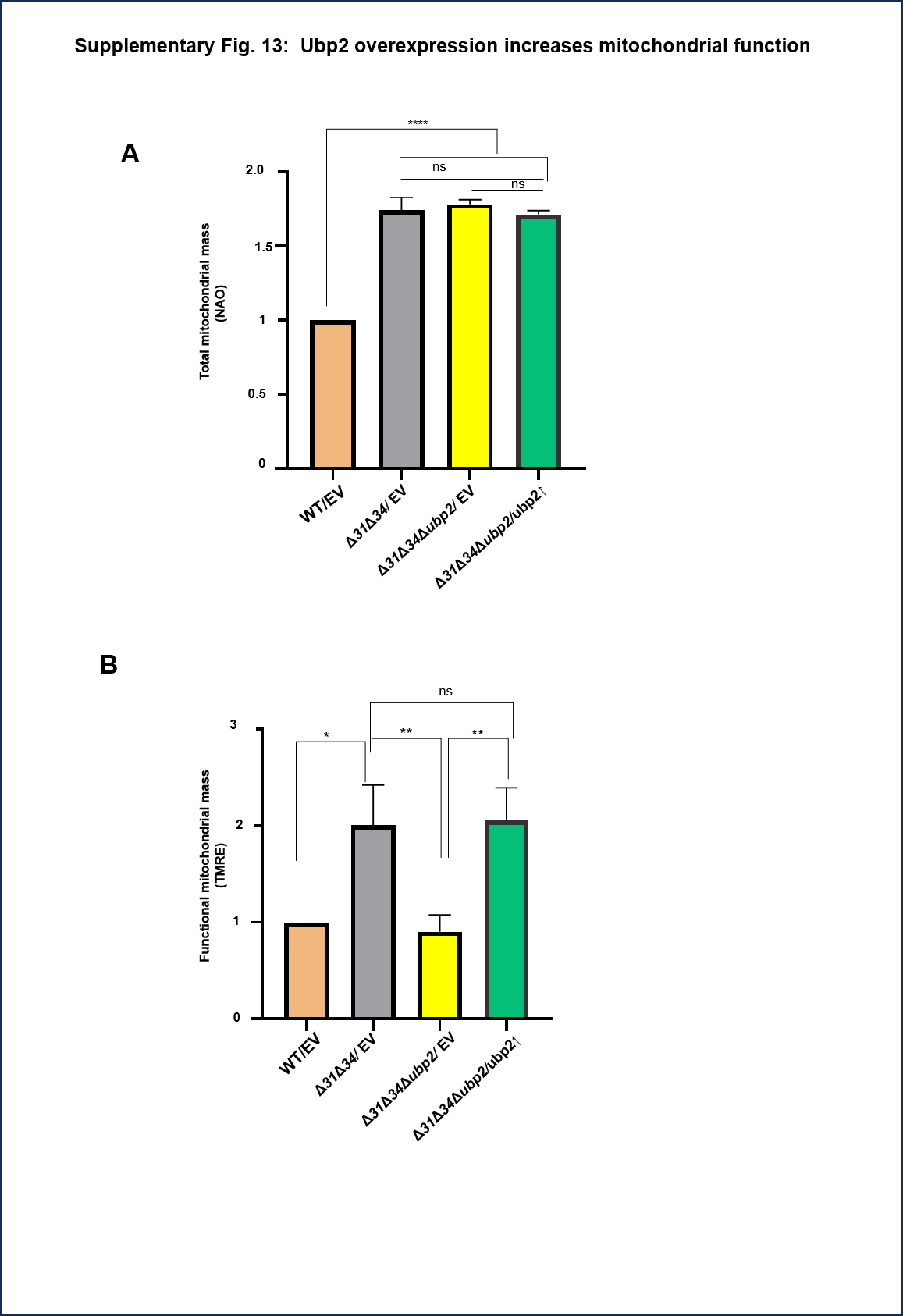

Supplement: S13 Fig — (A) Cells grown until the mid-log phase were subjected to NAO staining and quantified using BD FACS verse instrument. (B) Evaluation of the functional mitochondrial mass by flow cytometry. Cells grown until the mid-log phase were subjected to TMRE staining, a potentiometric dye, and acquired using BD FACS Verse. (TIFF) [file pgen.1011353.s013.tiff]

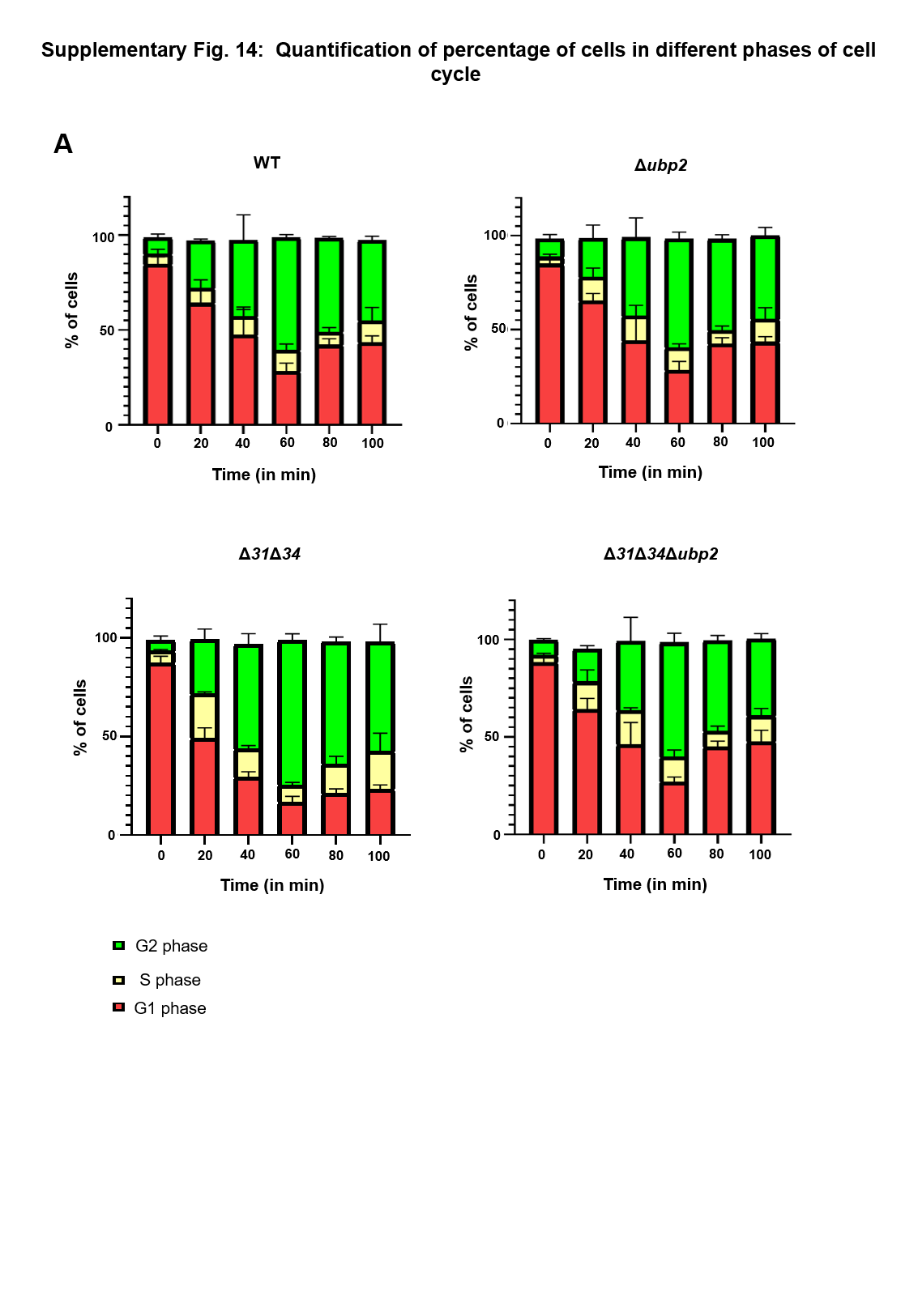

Supplement: S14 Fig — A graph was plotted depicting the percentage of cells at each time interval. (TIFF) [file pgen.1011353.s014.tiff]

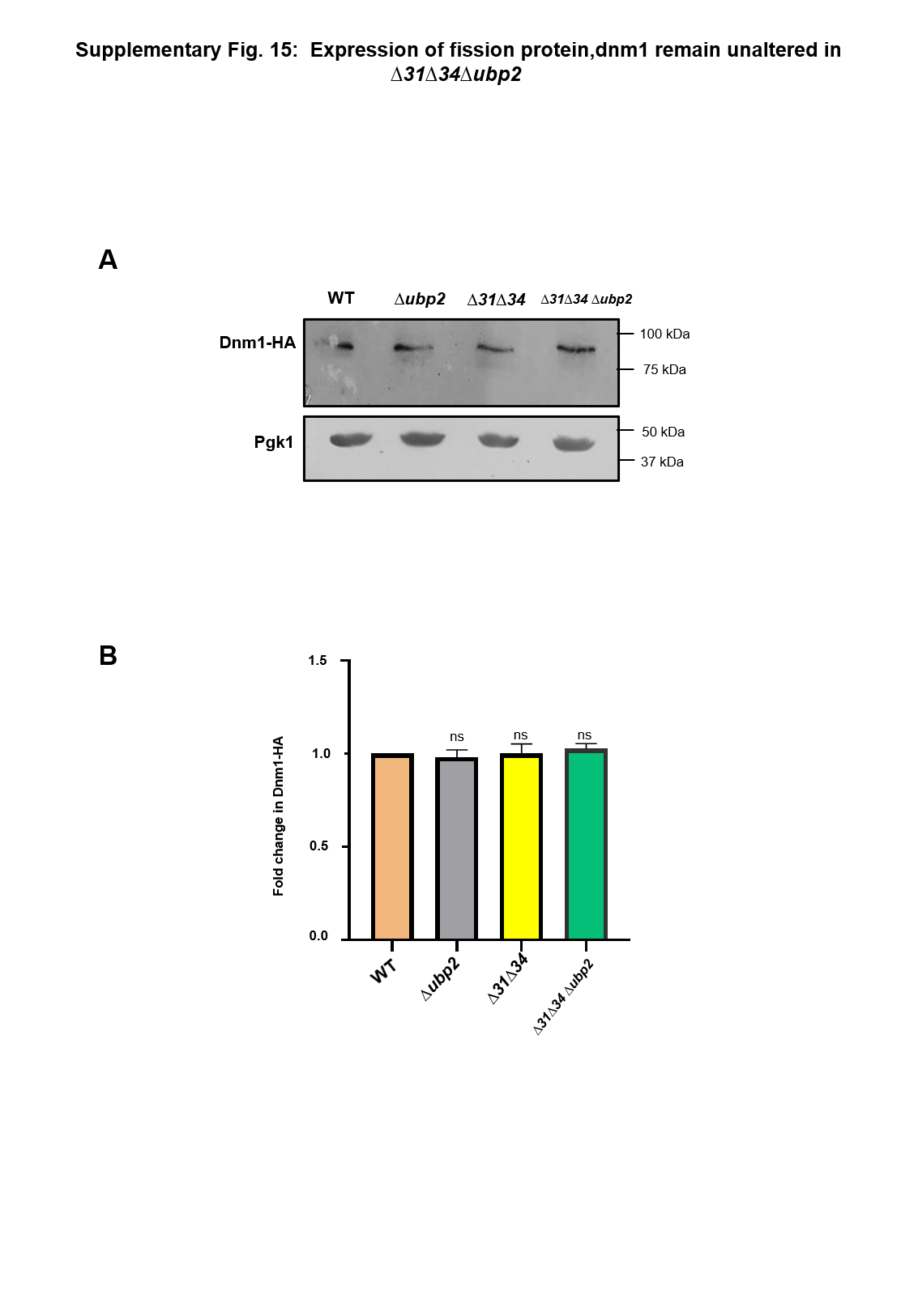

Supplement: S15 Fig — WT, ∆ubp2, ∆31∆34, ∆31∆34∆ubp2 were tagged with HA at the C-terminus of Dnm1 and lysates were subjected to Western blotting to check for difference in expression. (TIFF) [file pgen.1011353.s015.tiff]

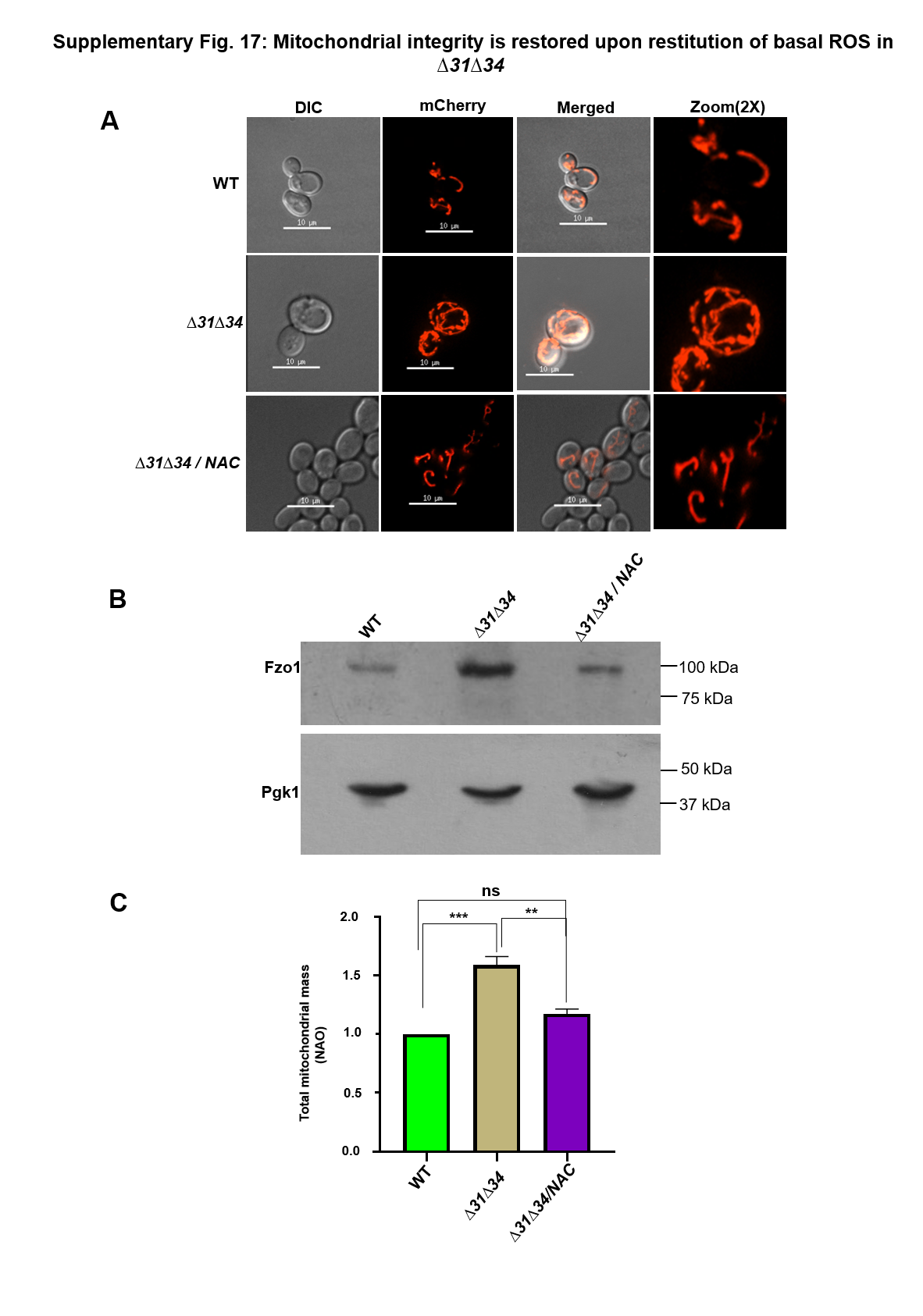

Supplement: S17 Fig — (A)The mitochondrial morphology of the indicated strains was visualised by utilising mts-mCherry constructs. Scale bar(10µm). (B)Fzo1 levels were probed by Western blotting. (C)The total mitochondrial mass was measured by NAO staining,followed by flow cytometry. (TIF) [file pgen.1011353.s017.tif]
