# Supplementary material for: Ubp2 modulates DJ-1-mediated redox-dependent mitochondrial dynamics in Saccharomyces cerevisiae
Source: PLoS Genet. 2025 Jul 3;21(7):e1011353. doi: 10.1371/journal.pgen.1011353 (PMC12251144; doi:10.1371/journal.pgen.1011353)
Supplement: S1 Table — (DOCX) [file pgen.1011353.s020.docx]

**List of primers used :**

| **S no.** | **Primer sequence ( 5’→ 3’)** | **Primer name** |
| --- | --- | --- |
| 1. | GTGGCTCAAAAATTGATGGTGGAAGAAATAAATTTAGACAT CGAT | Forward, Fzo1-HA tagging |
| 2. | GTATATTGATTTGAAAAGACCTCATATATTTACAAGAATATC TA | Reverse, Fzo1-HA tagging |
| 3. | CGTGTACGCATGTAACATTATAC | Fzo1-HA tag confirmation hphNT1 Forward |
| 4. | CTCATAAAGGAGGCAAA | Fzo1-HA tag confirmation, Reverse |
| 5. | GGTAATTAAAAAGAAAGCTTTTGTTCAAGGTTAAGAAGGTATAAGGAACGTACGCTGCAGGTCGAC | Ubp2 KO Forward |
| 6. | GGTACTTATGGCAATAGTGACATTTTACATAAACTCTTCATTGACTAAGAATCGATGAATTCGAGCTCG | Ubp2 KO Reverse |
| 7. | CTTCGTGGTCATCTCGTAC | Ubp2 KO confirmation natNT2 Forward |
| 8. | CTGTGGTTGATCCTGTGGGC | Ubp2 KO confirmation, Reverse |
| 9. | GATATTGAGCCATTGAAAAGAATTCTAAAGCGTACGCTGCAGGTCGAC | Forward, Ubp2-HA tagging |
| 10. | TTACATAAACTCTTCATTGACTAAGACTAATCGATGAATTCGAG | Reverse, Ubp2-HA tagging |
| 11. | CGTTATTGCTGATTTGCTC | Ubp2-HA tag confirmation natNT2 forward |
| 12. | AAAGGCTGCAACCCTTATTAGTAATATTCTGCGTACGCTGCAGGTCGAC | Forward, Dnm1-HA tagging |
| 13. | TAAGATCAAAAATGAGATGAATTATGCAATTAATCGATGAATTCGAGCTCG | Reverse, Dnm1-HA tagging |
| 14. | CGTGTACGCATGTAACATTATAC | Dnm1-HA tag confirmation hphNT1 forward |
| 15. | ATTGATGGAAGGCAATCTTC | Dnm1-HA tag confirmation, Reverse |
| 16. | CGGACTAGTATGCCGAACGAAGATAATGAAC | Forward, Ubp2 amplification |
| 17. | ACGCGTCGACCTAAGCGTAATCTGGAACATCGTATGGGTACTTTAGAATTCTTTTCAATGGC | Reverse, Ubp2 HA tag amplification |
|  | CGCGGATCCATGACCGCAAAGACTTTTCTACTACAGGC | Forward, Hap4 amplification |
|  | ACGCGTCGACTCAAGCGTAATCTGGAACATCGTATGGGTAAAATACTTGTACCTTTAAAAAATCGACATC | Reverse, Hap4 HA tag amplification |
|  | CGGACTAGTATGCCGAACGAAGATAATGAAC | Forward, Ubp2745S amplification |
|  | CCGCTCGAGCTACTTTAGAATTCTTTTCAATG | Reverse, Ubp2C745S amplification |

**List of strains used :**

| Strain | Genotype | Source |
| --- | --- | --- |
| BY4741 WT | *MATa his3Δ1 leu2Δ0 met15Δ0 ura3Δ0* | Open Biosystems |
| Δ*hsp31* | *MATa his3Δ1 leu2Δ0 met15Δ0 ura3Δ0* Δ*hsp31::KanMX4* | (Bankapalli et al., 2015) [1] |
| Δ*hsp32* | *MATa his3Δ1 leu2Δ0 met15Δ0 ura3Δ0* Δ*hsp32::hphNT1* | (Bankapalli et al., 2015) [1] |
| Δ*hsp33* | *MATa his3Δ1 leu2Δ0 met15Δ0 ura3Δ0* Δ*hsp33::hphNT1* | (Bankapalli et al., 2015) [1] |
| Δ*hsp34* | *MATa his3Δ1 leu2Δ0 met15Δ0 ura3Δ0* Δ*hsp34::URA3* | (Bankapalli et al., 2015) [1] |
| Δ*31*Δ*34* | *MATa his3Δ1 leu2Δ0 met15Δ0 ura3Δ0* Δ*hsp31::KanMX4;* Δ*hsp34::URA3* | (Bankapalli et al., 2015) [1] |
| Δ*ubp2* | *MATa his3Δ1 leu2Δ0 met15Δ0 ura3Δ0* Δ*ubp2::NAT* | This study |
| Δ*31*Δ*ubp2* | *MATa his3Δ1 leu2Δ0 met15Δ0 ura3Δ0* Δ*hsp31::KanMX4;* Δ*ubp2::NAT* | This study |
| Δ*34* Δ*ubp2* | *MATa his3Δ1 leu2Δ0 met15Δ0 ura3Δ0* Δ34*::URA3;* Δ*ubp2::NAT* | This study |
| Δ*31*Δ*34*Δ*ubp2* | *MATa his3Δ1 leu2Δ0 met15Δ0 ura3Δ0* Δ*hsp31::KanMX4;* Δ*hsp34::URA3;* Δ*ubp2::NAT* | This study |
| Δ*bar1* | *MATa his3Δ1 leu2Δ0 met15Δ0 ura3Δ0* Δbar1*::LEU2* | (Bankapalli et al., 2020) [2] |
| Δ*31*Δ*34*Δ*bar1* | *MATa his3Δ1 leu2Δ0 met15Δ0 ura3Δ0* Δ*hsp31::KanMX4;* Δ*hsp34::URA3;*Δbar1*::LEU2* | (Bankapalli et al., 2020) [2] |
| Δ*31*Δ*34* Δ*ubp2*Δ*bar1* | *MATa his3Δ1 leu2Δ0 met15Δ0 ura3Δ0* Δ*hsp31::KanMX4;* Δ*hsp34::URA3;* Δ*ubp2::NAT* Δbar1*::LEU2* | This study |
| WT/Ubp2-HA | *MATa his3Δ1 leu2Δ0 met15Δ0 ura3Δ0 Ubp2-HA::NAT* | This study |
| Δ*31*Δ*34*/ Ubp2-HA | *MATa his3Δ1 leu2Δ0 met15Δ0 ura3Δ0* Δ*hsp31::KanMX4;* Δ*hsp34::URA3*; *UBP2-HA::NAT* | This study |
| WT/Fzo1-HA | *MATa his3Δ1 leu2Δ0 met15Δ0 ura3Δ0 FZO1-HA::NAT* | This study |
| ∆*ubp2*/Fzo1-HA | *MATa his3Δ1 leu2Δ0 met15Δ0 ura3Δ0 FZO1-HA::NAT* | This study |
| Δ*31*Δ*34*/Fzo1-HA | *MATa his3Δ1 leu2Δ0 met15Δ0 ura3Δ0 FZO1-HA::NAT* | This study |
| Δ*31*Δ*34*∆*ubp2*/Fzo1-HA | *MATa his3Δ1 leu2Δ0 met15Δ0 ura3Δ0 FZO1-HA::NAT* | This study |
| WT/OM45-GFP | MATa his3∆1 leu2∆0 met15∆0 ura3∆0 OM45- GFP::hphNT1 | (Bankapalli et al., 2020) [2] |
| Δ*ubp2*/OM45-GFP | MATa his3∆1 leu2∆0 met15∆0 ura3∆0 Δ*ubp2::NAT*;OM45- GFP::hphNT1 | This study |
| ∆31∆34/OM45-GFP | MATa his3∆1 leu2∆0 met15∆0 ura3∆0 ∆hsp31::KanMX4; ∆hsp34::URA3; OM45-GFP::hphNT1 | (Bankapalli et al., 2020) [2] |
| ∆31∆34∆*ubp2*/OM45-GFP | MATa his3∆1 leu2∆0 met15∆0 ura3∆0 ∆hsp31::KanMX4; ∆hsp34::URA3; Δ*ubp2::NAT* ;OM45-GFP::hphNT1 | This study |
| ∆atg32/OM45-GFP | MATa his3∆1 leu2∆0 met15∆0 ura3∆0 ∆atg32:: HIS3; OM45-GFP::hphNT1 | (Bankapalli et al., 2020) [2] |
| WT/Dnm1-HA | MATa his3∆1 leu2∆0 met15∆0 ura3∆0 DNM1-HA::KanMX4 | (Bankapalli et al., 2020) [2] |
| Δ*ubp2*/Dnm1-HA | MATa his3∆1 leu2∆0 met15∆0 ura3∆0 Δ*ubp2::NAT*;DNM1-HA::KanMX4 | This study |
| ∆*31*∆*34*/Dnm1-HA | MATa his3∆1 leu2∆0 met15∆0 ura3∆0 ∆hsp31::KanMX4; ∆hsp34::URA3; DNM1::hphNT1 | (Bankapalli et al., 2020) [2] |
| ∆*31*∆*34* Δ*ubp2*/Dnm1-HA | MATa his3∆1 leu2∆0 met15∆0 ura3∆0 ∆hsp31::KanMX4; ∆hsp34::URA3; Δ*ubp2::NAT;* DNM1::hphNT1 | This study |

**References:**

1. Bankapalli K, Saladi S, Awadia SS, Goswami AV, Samaddar M, D'Silva P. Robust glyoxalase activity of Hsp31, a ThiJ/DJ-1/PfpI family member protein, is critical for oxidative stress resistance in Saccharomyces cerevisiae. J Biol Chem. 2015 Oct 30;290(44):26491-507. doi: 10.1074/jbc.M115.673624. Epub 2015 Sep 14. PMID: 26370081; PMCID: PMC4646309.
2. Bankapalli K, Vishwanathan V, Susarla G, Sunayana N, Saladi S, Peethambaram D, D'Silva P. Redox-dependent regulation of mitochondrial dynamics by DJ-1 paralogs in Saccharomyces cerevisiae. Redox Biol. 2020 May;32:101451. doi: 10.1016/j.redox.2020.101451. Epub 2020 Feb 7. PMID: 32070881; PMCID: PMC7026286.
