# Supplementary material for: Ubp2 modulates DJ-1-mediated redox-dependent mitochondrial dynamics in Saccharomyces cerevisiae
Source: PLoS Genet. 2025 Jul 3;21(7):e1011353. doi: 10.1371/journal.pgen.1011353 (PMC12251144; doi:10.1371/journal.pgen.1011353)
Supplement: S2 Table — (DOCX) [file pgen.1011353.s021.docx]

| **Antibody name** | **Company/Gifted by** | **Catalog Number** | **References**  **(if applicable)** |
| --- | --- | --- | --- |
| Monoclonal anti-HA tag antibody | Sigma-Aldrich | SAB2702217 | - |
| Anti-Ubiquitin antibody | Novus Biologicals | NB300-130 | - |
| Anti-Green Fluorescent Protein (GFP) antibody | Sigma-Aldrich | G1546 | - |
| Amersham ECL Mouse IgG antibody | Cytiva | NA931V | - |
| Anti-Rabbit IgG (whole molecule)–Peroxidase antibody | Sigma-Aldrich | A0545 | - |
| Anti-Fzo1 antibody | Prof. Mafalda Escobar-Henriques, Centre for Molecular Medicine, Cologne, Germany | - | Simões, Tânia *et al*., 2018 [1] |
| Anti-Pgk1 antibody | Prof. Pundi N Rangarajan, Department of Biochemistry, Indian Institute of Science, Bengaluru | - | Dey, Trishna, *et al.*,2018 [2] |
| Anti-Tim44 antibody | Prof. Elizabeth Craig, Department of Biochemistry, University of Wisconsin-Madison | - | D'Silva, Patrick  et al.,2004 [3] |

**List of antibodies used in the study:**

**references**

**References**

1. Simões T, Schuster R, den Brave F, Escobar-Henriques M. Cdc48 regulates a deubiquitylase cascade critical for mitochondrial fusion. Elife. 2018 Jan 8;7:e30015. doi: 10.7554/eLife.30015. PMID: 29309037; PMCID: PMC5798933.
2. Dey T, Krishna Rao K, Khatun J, Rangarajan PN. The nuclear transcription factor Rtg1p functions as a cytosolic, post-transcriptional regulator in the methylotrophic yeast *Pichia pastoris*. J Biol Chem. 2018 Oct 26;293(43):16647-16660. doi: 10.1074/jbc.RA118.004486. Epub 2018 Sep 5. PMID: 30185617; PMCID: PMC6204902.
3. D'Silva P, Liu Q, Walter W, Craig EA. Regulated interactions of mtHsp70 with Tim44 at the translocon in the mitochondrial inner membrane. Nat Struct Mol Biol. 2004 Nov;11(11):1084-91. doi: 10.1038/nsmb846. Epub 2004 Oct 17. PMID: 15489862.
