# Supplementary material for: Ubp2 modulates DJ-1-mediated redox-dependent mitochondrial dynamics in Saccharomyces cerevisiae
Source: PLoS Genet. 2025 Jul 3;21(7):e1011353. doi: 10.1371/journal.pgen.1011353 (PMC12251144; doi:10.1371/journal.pgen.1011353)
Supplement: S3 Table — (DOCX) [file pgen.1011353.s022.docx]

**List of reagents used in the study:**

| **Antibody name** | **Company/Gifted by** | **Catalog Number** |
| --- | --- | --- |
| Protein G Sepharose 4 Fast Flow beads | Cytiva | 17061801 |
|  |  |  |
| Nonyl Acridine Orange (Acridine Orange 10-Nonyl Bromide) | Thermo Fisher Scientific | A1372 |
| JC-1 dye | Thermo Fisher Scientific | T3168 |
| H_2_DCFDA dye | Sigma-Aldrich | D6883 |
| ATP assay kit | Sigma-Aldrich | MAK190 |
| Alpha factor | Sigma-Aldrich | T6901 |
| Propidium Iodide | Thermo Fisher Scientific | P3566 |
| MitoSox dye | Thermo Fisher Scientific | M36005 |
| H_2_O_2_ | Merck | 88597 |
| Monochlorobiamine (MCB) | Sigma-Aldrich | 69899 |
| GSH/GSSG assay kit | Sigma-Aldrich | MAK440 |
| TMRE | Thermo Fisher Scientific | T669 |
| Digitonin | Sigma-Aldrich | D5628 |
