# Supplementary material for: Ubp2 modulates DJ-1-mediated redox-dependent mitochondrial dynamics in Saccharomyces cerevisiae
Source: PLoS Genet. 2025 Jul 3;21(7):e1011353. doi: 10.1371/journal.pgen.1011353 (PMC12251144; doi:10.1371/journal.pgen.1011353)
Supplement: Attachment — Submitted filename: Author response for Reviewers_240425.pdf [file pgen.1011353.s054.pdf]

Reviewer 1:

General comments:

Comment 1:

This is a potentially interesting manuscript that characterizes several mitochondria-related phenotypes in mutants lacking yeast DJ-1 proteins. The authors have found a genetic link between Hsp31/34 and the Ubp2 cysteine-dependent deubiquitinase. They show that loss of UBP2 restores some previously described phenotypes in strains lacking Hsp31/34. Whilst the authors have shown that loss of Ubp2 suppresses a number of phenotypes in hsp31, 34 mutants, it is not clear that this represents a physiologically significant crosstalk in wild-type cells. For example, loss of Ubp2 has similar effects in both wild-type and Δ31Δ34 strains for a number of the phenotypes examined so the authors may simply be reporting on what loss of Ubp2 causes rather than specificity for Δ31Δ34 strains. It is interesting though, since loss of Ubp2 clearly rescues the respiratory growth phenotype of Δ31Δ34 strains.

Author response

We thank the reviewer for appreciating our current findings.

Most notably, Ubp2 deletion restores the mitochondrial-specific defects associated with the deletion of DJ-1 paralogs. Moreover, Ubp2 restores the phenotype only upon the combinatorial deletion of Hsp31 and Hsp34, suggesting a specific interaction with the DJ-1 paralogs. Therefore, the current findings thus integrate the crosstalk between deubiquitinase and DJ-1 paralogs in regulating cellular health for the first time.

Comment 2:

The manuscript presents a complicated data set, which at present are largely correlative. As outlined below, some conclusions are somewhat over-stated in the Discussion based on the current findings and more data are required to be so definitive. Other experiments require further analysis and controls as outlined below.

Author response

Thank you for your valuable suggestions. We have now incorporated the changes, including image quality, statistical analysis, and new experimental findings.

Recommendations for the authors

Specific Comments:

Comment 1:

Fig. 1A – images are poor quality, particularly for SC glycerol growth at 37C. Given that the same data is shown in Fig. S1 and it is much clearer, it should be replaced in the main manuscript figure.

Author response

Thank you for your suggestion. We have incorporated the changes in the revised version of the manuscript. In the revised version, we have changed Fig. S1 to **Fig. 1A**.

Comment 2:

Figure 1B shows that Ubp2-HA is increased in Δ31Δ34 strains whereas there does not appear to be any difference in Ubp2-Ha for the mid-log phase cells shown in Fig. S2. It is not stated what growth phase was used for Fig. 1B but presumably it should have been mid-log phase to be consistent with the spot tests. This should be clarified, especially what is meant by mid-log phase (6h) and late-log phase (12h) which should be defined on growth curves. The Discussion states that DJ-1 paralogs regulate the growth phase dependent expression of Ubp2 but much more careful experiments are required to reach such a strong a conclusion. This is important since it is the only direct evidence that Hsp31, 34 affects Ubp2.

Author response

Thank you for the valuable comments. We have performed a growth curve assay and integrated the data into the manuscript. Kindly refer to Fig. S1A.

Indeed, the spot assays have been performed with cells in the mid-log phase. At the same time, the spot assay was performed in a non-fermentable carbon source (glycerol) to ascertain mitochondrial respiration's role in cells lacking DJ-1 paralogs. Interestingly, Ubp2 was found to have differential expression in Δ*31*Δ*34* only at the late log phase in dextrose as the carbon source. Moreover, DJ-1 paralogs have previously been reported to be necessary for metabolic reprogramming during diauxic shift and cellular survival at the stationary phase (**Miller-Fleming, Leonor *et al.*; PMID : 24706893**).

In the absence of DJ-1 paralogs, several metabolic responses are affected due to perturbed expressions of several genes during the diauxic shift. Given the altered expression of genes involved in oxidative stress response in the absence of DJ-1 paralogs, the expression of redox-sensitive Ubp2 is altered at the late log phase, as presented in **Fig.1B and Fig.S1**.

Comment 3:

Figure 2D – Given the differential effects of Ubp2 overexpression in wild-type and Δ31Δ34 strains it is important to confirm that Ubp2 is expressed to similar levels in both strains. This control should be straight-forward using the HA tag.

Author response

Thank you for the valuable suggestion. Immunoblotting was performed to probe for Ubp2-HA expression in the overexpression plasmids across all the strain backgrounds. The data is now represented in **Fig S2** in the revised version.

Comment 4:

The mitochondrial integrity assay shown in Fig. 2A needs more explanation. Does

intermediate mean a mixture of fragmented and tubular mitochondria within cells? It would help to provide some illustrative diagrams to differentiate what is meant by fragmented, intermediate and tubular. It would be better to present the data as a percentage, presumably from triplicate experiments although this is not stated. The authors should explain how they scored the different morphologies plus discuss any potential physiological significance.

Author response

Thank you for the response. An illustration depicting the different mitochondrial morphologies has been incorporated in **Fig. 2A.** The assay has now been changed to a percentage of cells. Kindly refer to **Fig. 2B** in the revised version.

The analysis of mitochondrial morphologies was done using **ImageJ** software, and the scoring analysis is incorporated in the "**Materials and methods section**."

The physiological significance of cells adapting to different mitochondrial structures is incorporated in the "**Introduction**" section.

Comment 5:

The mitophagy assay shown in Fig. 3 needs more explanation. What do the various smaller OM45-GFP fragments correspond to? Why are there 3 different size fragments. The description of this data in the text is quite subjective and it would benefit from quantification and an indication of reproducibility.
Fig. 3B legend – what do the dashed boxes and arrows indicate

Author response

Thank you for the queries and suggestions. The different fragments of GFP are perhaps due to differential cleavage of GFP processing. Vacuolar proteases process the tagged [OM45-GFP(75kDa)] upon mitophagy induction. The GFP is processed into multiple fragments (~20kDa -27kDa) by proteolytic cleavage of fusion proteins, thus leading to immunodetection of the multiple cleaved GFP fragments.

We apologize for not representing the data as quantified. The extent of GFP processing across the strains has now been integrated into **Fig. S4B.** The legend of **Fig. 3B** has been changed to explain the vacuole-dependent processing of mitochondria.

Comment 6 :

The quantifications shown in Fig. 4D does not appear to match the western blots shown in Fig. 4C. For example, a very strong Fzo1 signal is detected in the ubp2 mutant at T = 0 and there is essentially no signal detected at 90 min. However, the quantification suggests that just 50% of Fzo1 remains at 90 min. The quantification also lacks statistical analysis. For this blot and others, it is not stated whether signals were normalized to loading controls as part of the quantitative analysis.

Following on from these experiments the Discussion states that the increased expression of Ubp2 contributes to the increased stability of Fzo1 in Δ31Δ34 cells – In

order to reach this strong conclusion, the authors need to demonstrate that altering Ubp2 levels in a wild-type strain using their overexpression construct affects Fzo1 stability and other read-outs of mitochondrial function, dependent on the presence of Hsp31/34.

Author response

We apologize for the mistake in quantification. **Fig. 4C** has now been corrected, and statistical analysis has been incorporated (two-way ANOVA). The signals were normalized to loading controls to quantify all the blots.

Thank you for the response. Indeed, Ubp2 overexpression in WT background leads to hyperfusion of mitochondria and elevated expression of Fzo1, highlighting its role in Fzo1 stabilization.
The new results have been incorporated in **Fig. S5** in the revised version.

Comment 7:

Fig. 5 and line 332 The authors suggest there is a cell cycle arrest at the G2/M phase in the Δ31Δ34 mutant. Presumably they mean there is a delay in the cell cycle at this stage. To aid a more meaningful comparison, this data would benefit from quantitation perhaps by calculating the length of time each strain spends in the different stages of the cell cycle.

Author response

Thank you for the valuable suggestion. Line 364 (previously 332) has been rephrased to "Δ*31*Δ*34* strain exhibited slower progression as more than 50% of the cells continued to be at the G2/M phase (from t=80 min to t=100 min)*"*. The percentage of cells in the G1, S, and G2 phases has also been plotted as a function of time. Kindly refer to **Fig. S11** in the revised manuscript.

Comment 8:

As discussed by the authors, it is the GSH to GSSG ratio that is the crucial indicator for alterations in redox state and glutathione metabolism. Simply measuring GSH levels using monochlorobimaine is not sufficient to comment on changes in redox state. These assays should be repeated using approaches that measure both GSH and GSSG levels preferably using fluorescent reporter probes that report on the redox state of the different cytoplasmic and mitochondrial glutathione pools, rather than in whole cell extracts.

Author response

We thank the reviewer for the suggestion. As suggested, GSH and GSSG levels have been represented as ratios in the mitochondrial and cytoplasmic extracts. Kindly refer to **Fig. 7D and Fig. 7E** in the revised manuscript.

Reviewer 2

General Comments:

Comment 1:

The manuscript „Ubp2 modulates DJ-1-mediated redox-dependent mitochondrial dynamics in Saccharomyces cerevisiae“ by Sananda Biswas and Patrick D'Silva describes a functional link between the deubiquitinase Ubp2 and DJ-1-like Hsp31 paralogues in yeast. The study provides novel insights on how Ubp2 and Hsp31 paralogues regulate mitochondrial fission/ fusion processes and give evidence for involvement of ROS production and mitophagy. Even though the manuscript delivers novel insights, some fundamental issues need to be addressed as part of a major revision. In general the statisics need to be improved and clarified for each figure. The authors tend to over-interprete the data based on single images without robust statistical evidence. The authors should only make claims where appropriate (see list). The major and minor points to be addressed are listed as follows.

Author response

Thank you for your valuable input and suggestions for making our manuscript better. The statistics on each figure have been incorporated and interpreted accordingly in the revised manuscript. We curtailed our interpretation as per your suggestions.

Recommendations for the authors

Comment 1:

1) The authors need to give information on the statistical tests performed for each figure individually. The general statistics statement is not sufficient. Some statistical tests are missing and need to be conducted.

Author response

We apologize for not mentioning the statistical tests performed. We have now added the appropriate statistical tests for every figure.

Comment 2:

2) Figure 3A: The authors need to quantify the ratios of free GFP to OM34-GFP + free GFP (at least triplicates) and show additional replicates as supplemental data. The authors should be careful with their claims. It seems like the expression level of OM45-GFP is reduced in Δubp2.
Why are there additional bands in Δubp2 (below 20 kDa)?

Author response

Thank you for your valuable suggestions. We quantified the blots (multiple sets together with controls) and presented the graph in **Fig. S4.**

The expression level of OM45-GFP remains similar in Δubp2 compared to the control confirmed by triplicate experiments, represented in **Fig. S4.**
Due to the differential cleavage of GFP, multiple fragments were observed in Δubp2 and other strains. Vacuolar proteases digest OM45-GFP (75 kDa) when mitophagy is induced. Through proteolytic cleavage of fusion proteins, GFP is cleaved into numerous fragments (~20kDa–27kDa), which are immunodetected by anti-GFP antibodies.

Comment 3:

3) Lines 273-277: It is not adequate to make such claims based on single images without any quantitative assessment. At least for Δ31Δ34 and Δ31Δ34 Δubp2 a co-staining for vacuoles should be performed to properly identify vacuolar GFP signals.

Author response

Thank you for your valuable suggestion. We have stained the cells undergoing mitophagy with FM4-64 dye, a vacoule staining dye. Please refer to **Fig. 3B**.

Minor points:

Comment 1:

1. Data availability statement indicates that data will be accessible, but no precise platform or link is given. This needs to be completed.

Author response

Thank you for the response. The raw data files have now been included in the supplementary and made available to the reviewers.

Comment 2:

2. Line 169: I do not observe growth impairment of Δubp2 at 24°C on dextrose media. Please rephrase accordingly.

Author response

We thank the reviewer for the response. In the revised manuscript, we have changed the statement to the following: 'The deletion of Ubp2 (Δubp2) leads to an impairment in the growth in both complete (S.C. Dextrose) and non-fermentable media (S.C. Glycerol) at a non-permissive temperature of 37 °C.'

Comment 3:

3. Line 169-174: No hsp single mutants are shown in Fig. 1A. The reference should be made to Fig. S1.

Author response

We apologize for the mistake. Since Fig. S1A will be represented as Fig. 1A in the revised manuscript, the figure's citation has not been changed.

Comment 4:

4. Line 170: Δ31 show only a very weak decrease in growth.

Author response

We apologise for the misinterpretation. The statement has been rephrased as "among the four Hsp31 paralogs, only the cells lacking Hsp31(Δ*31*) exhibit a slight reduction in growth in glycerol at a non-permissive temperature of 37°C".

Comment 5:

5. Figure S1: The temperature needs to be properly indicated in columns.

Author response

We apologize for the mistake. Fig. S1A will be represented as Fig. 1A, and the temperatures have been indicated.

Comment 6:

6. Figure 1 C: Which statistical test was performed to calculate the p-value?

Author response

We thank the reviewer for the valuable input. An unpaired student t-test was performed, and the statistical methods have now been represented in the revised manuscript.

Comment 7:

7. Figure S2: The bars indicating 6h and 12h are not properly positioned in the figure. Line 188-190: To claim that Ubp2-HA levels are significantly changed in wt requires quantitative western blotting with suitable statistics.

Author response

We apologize for the mistake. The figure has been corrected and quantified as depicted in **Fig. S1C**.

Comment 8:

8. Figure 2: It is required to mention the statistical tests (ANOVA?) which were performed. How was the classification of mitochondria made?

Author response

We apologize for not mentioning the statistical tests performed. One-way ANOVA (non-parametric) was utilized to score the mitochondrial morphologies. An illustration depicting different mitochondrial forms is now incorporated in **Fig. 2A**. Further, the

mitochondrial morphologies were scored using **ImageJ** and discussed in the "**Materials and Methods section**." Briefly, after setting the scale, thresholding for mitochondrial morphology was done and cells with mitochondria having size in the range of 4-10 pixels were labeled as 'intermediate' while the ones above this value range were labeled as 'tubular'. Cells below the range of 4 pixels were defined to have 'fragmented' mitochondria.

Comment 9:

9. Line 226: The abbreviation NAO needs to be introduced here.
Line 228 and 231: The reference should be Fig. 2C.
Line 236/239: The reference should be Fig. 2D.
Line 237: A citation is missing.
Line 245: A space is missing between "defects(Fig. 2E)"

Author response
We apologize for the errors. The mistakes have been rectified in the revised manuscript.

Comment 10:

10. Lines 263-265: "Δ31Δ34 showed slower mitophagy induction after 54 h of growth in glycerol due to the presence of hyperfused mitochondrial structures" The authors do not show evidence for the causal link and should rephrase their claim accordingly.

Author response
We thank the reviewer for the valuable technical input. The statement has now been rephrased as '*Δ31Δ34* exhibits comparable mitophagy rate to WT, albeit at a lower processing efficiency. However, *Δ31Δ34* showed slower mitophagy induction possibly due to the presence of hyperfused mitochondrial structures (Fig. 3A & S4B).'

Comment 11:

11. Line 275: "faster" instead of "fater"

Author response
We apologize for the errors. The mistakes in the manuscript have been rectified.

Comment 12:

12. Figure 4: What kind of statistical test was performed?
Statistical analysis of 4D is missing (2-way ANOVA)

Author response
We apologize for not stating the statistics performed. Indeed, 2-way ANOVA was performed in **Fig.4** and has been included in the figure legend of the revised manuscript.

Comment 13:

13. Line 330: Cite Fig. 5
Line 340: "Microscopic analysis revealed that Δ31Δ34 has 30% of the cells exhibiting increased cell size than WT (more than 5-6 µm)" There is no figure supporting this statement. The authors should show the quantification as supplemental data.

Author response

We apologize for the mistake. The values have now been incorporated into the supplementary material. We have now changed the statement to provide a more meaningful interpretation. The statement has been changed to " Microscopic analysis revealed that Δ31Δ34 has cells with an average cell size of ~9 µm exhibiting increased cell size than WT (average cell size of 4-6 µm)"

Comment 14:

14. Line 347: "lelevls" to "levels"

15. Line 353: Introduce abbreviation for H2DCFDA.

16. Figure 6: Add note for statistics.
Line 358: These findings were further…
Line 369: "… the microscopic analysis was performed …
Line 376: Full stop is missing.
Line 406: Omit "with"
Line 411: To avoid confusion the authors should write "perturbation of Fzo1" instead of "perturbation of the fusion protein"
Line 433: Full stop is missing.

Author response

We apologize for the errors. The mistakes have been rectified in the revised manuscript.

Reviewer 3

Comment 1:

This manuscript addresses the relationship between the PARK7 paralogues Hsp31/34 and the USP10 paralogue Ubp2. In a previous study, the authors nicely showed that Hsp31 and Hsp34 are required for redox balance, mitochondrial respiration, mitochondrial morphology and cell-cycle progression. Simultaneous loss of Hsp31and Hsp34 causes a reduction in GSH levels and consequently oxidative stress, increased mitochondrial biogenesis, and mitochondrial mass, G2/M cell cycle arrest, and increased apoptosis, for the most part reverted by addition of the antioxidant NAC. Here, the authors convincingly show that ablation of the deubiquitinase Ubp2 suppresses all defects associated to loss of Hsp31/34. They also observe a correlation between Ubp2/Hsp31/34 and alterations in mitochondrial morphology and in the MFN1/MFN2 paralogue Fzo1, and propose a role of mitochondrial fusion in this process. These findings are novel and potentially very relevant. Rescue experiments and mechanistic aspects of this rescue should be substantiated, in particular investigating causal effects, as detailed below.

Author response
We thank the reviewer for critically analyzing the current study and highlighting the novelty of the present findings.

Main points

Comment 1:

1) The authors nicely discover a genetic interaction between Ubp2 and Hsp31/34. In this regard:

A) They should provide evidence that re-expression of Ubp2 rescues all hsp31 hsp32 phenotypes presented in this manuscript.

Author response
Thank you for the valuable suggestions. We have re-expressed Ubp2 in the triple deletion strain (*Δ31Δ34Δubp2*) and analyzed for the mitochondrial respiration competency **(Fig. 1D)**. Besides, the immunoblotting and cycloheximide chase assay also suggests stabilized expression of Fzo1 upon overexpression of Ubp2 **(Fig. S6).** Further, phenotypic analysis was performed to check for the response of cells to the treatment of H_2O_2 upon Ubp2 overexpression **(Fig. S7A).** DCFDA staining was also performed, and the microscopic analysis corroborated the observed phenotype **(Fig. S7B).** Finally, the mitochondrial network morphology was analyzed whereby Ubp2

overexpression in Δ*31*Δ*34*Δ*ubp2* reinstated the hyperfused mitochondrial structures, resembling Δ*31*Δ*34* **(Fig. S8).**

Comment 2:

B) Moreover, is this rescue dependent on the catalytic activity of Ubp2?

Author response

We thank the reviewer for the valuable suggestion. A catalytically dead mutant of Ubp2 was constructed by mutating Cysteine to Serine mutation at the 745 residues (**Lam, Mandy H Y et al.** PMID:**19165343**), (**Santos, Clara M** ***et al.*****PMID: 39384040**). We visualized the mitochondrial network morphology upon overexpression of Ubp2 C745S in Δ*31*Δ*34*Δ*ubp2*, and the mitochondrial structures resembled WT **(Fig. S8, panel 4).** This suggests that the catalytic cysteine is indispensable for maintaining the function of mitochondrial fusion.

Comment 3:

C) If yes, how do the authors explain that UBP2 deletion rescues hsp31 hsp32 phenotypes, given that Ubp2 is expected to have reduced activity in hsp31 hsp32 cells. This prediction is because hsp31 hsp32 cells have increased ROS levels (PMID: 37548361and 32070881) and oxidative stress inactivates the catalytic activity of Ubp2 (PMID: 25622294).

Author response

Thank you for the response. Yes, the catalytic activity of Ubp2 is necessary for mediating the rescue observed in Δ*31*Δ*34*Δ*ubp2*. As reported previously, Ubp2 is inactivated reversibly in response to H_2O_2 stress (0.6mM- 2.5mM) (**Silva, Gustavo M et al.**PMID: **25622294**) as an extraneous stressor. However, in the absence of DJ-1 paralogs, there is an inherent increase in the ROS levels, maybe less than what we used for the experimental condition that we genuinely believe. At this, lower ROS levels in the absence of DJ-1 paralogs plausibly do not inactivate the catalytic cysteine of Ubp2. Moreover, Ubp2 is expressed more in Δ*31*Δ*34* than WT **(Fig 1B),** leading to higher activity causing hyperfusion of mitochondrial structures without DJ-1 paralogs.

Comment 4:

D) Along this line, repressed activity of Ubp2 is expected to cause mitochondrial fragmentation, but the hsp31 hsp32 cells have increased mitochondrial tubulation. How do the authors explain this?

Author response

Thank you for the critical response. Ubp2 is expressed more in the absence of DJ-1 paralogs and is plausibly not inactivated by intrinsic ROS. Thus, enhanced expression of Ubp2 stabilizes Fzo1 and causes increased mitochondrial tubulation.

Comment 5:

2) Related to point 1), can it be that catabolic repression caused by growth in glucose is absent in hsp31 hsp32 cells? Consistently, mitochondrial morphology of the hsp31 hsp32 cells intriguingly resembles WT cells in post-diauxic shift phase (PDS). Therefore, the authors should test if PDS cells resemble hsp31 hsp32 cells in cell cycle progression, mitophagy, ROS levels or growth resistance to H2O2. If yes, are these effects in PDS cells rescued by deletion of UBP2?

Author response

Thank you for your critical response. The cells in post-diauxic shift were phenotypically assessed in the presence of H_2O_2. However, the PDS cells exhibit resistance to oxidative stress **(Fig. S9)**. Cell cycle progression and mitophagy assessment could not be performed due to the technical drawbacks in designing the experiments. For instance, in the current study, the cells were arrested in the G1 phase to synchronize the culture. However, synchronization of cells in PDS could not be performed as PDS cells were not responsive to the alpha factor.

Similarly, for mitophagy induction, cells in the mid-log phase are transferred from dextrose-containing media to glycerol, and samples are collected over the indicated time points. Hence, cells in post-diauxic shift could not be analyzed for mitophagy. From the experiment performed, even though Δ*31*Δ*34* resembles the mitochondrial morphology of PDS cells, the PDS cells are oxidative stress resistant. Previous reports also indicate stress resistance to hydroperoxides in cells undergoing post-diauxic shift (**Maris, A F et al.** PMID: **11409175).**

Comment 6:

3) Related to point 2) is the rescue of respiration, mitochondrial mass, cell cycle arrest and ROS levels observed in ubp2 hsp31 hsp32 cells rather due to effects in the TCA and ETC pathways? The authors could follow an approach similar to https://doi.org/10.1101/2024.07.09.602707. For example, is the rescue dependent on the carbon source? What happens upon deletion of RTG1 or Hap4 overexpression?

Author response

Thank you for your valuable input. We over-expressed Hap4 in the background of Δ*31*Δ*34*and Δ*31*Δ*34*Δ*ubp2* to phenotypically assess the growth of the strains in different carbon sources as suggested **(Fig. S10)**. However, Hap4 over-expression showed a slight improvement in the growth of Δ*31*Δ*34*Δ*ubp2 in both glycerol and dextrose*, highlighting the minor effect of TCA/ETC pathways in regulating mitochondrial dynamics. However, more detailed studies are needed in this direction which, we believe that it is beyond the scope of the current manuscript.

Comment 7:

4) hsp31 hsp32 cells have altered levels and stability of Fzo1. How do these chaperones affect Fzo1? The authors should test if this is caused by an increase in mitochondrial biogenesis.

Of note, in PMID: 32070881 they observe that hsp31 hsp32 cells have increased transcriptional of all 3 major mt dynamics proteins: Dnm1, Mgm1 and Fzo1, and very comparable increases in their respective protein levels. Consistently, in this same manuscript, they show that hsp31 hsp32 cells have increased mitochondrial mass

and increased GSSG levels, thus altering the GSH/GSSG ratio. Reduction in GSH is an indicator of oxidative stress and they could revert increased mitochondrial mass with the antioxidant NAC.

Author response

We thank the reviewer for the suggestion. We thank the reviewer for the critical assessment.Indeed, hyperfusion caused due to stabilized Fzo1 also causes increased biogenesis as indicated by increment in expression of mitochondrial proteins (Tom70,Tim44, Tim23) as mentioned in **Bankapalli, Kondalarao *et a*l. ,2020** (PMID:**32070881**). The GSH and GSSG levels have been represented as ratios in the mitochondrial and cytoplasmic extracts. Kindly refer to **Fig. 7D and Fig. 7E** in the revised manuscript.

Comment 8:

5) They infer that UBP2 deletion rescues the cellular defects of hsp31 hsp32 cells due to the ubiquitination, stability and steady state levels of Fzo1. However, besides presenting correlative data, they do not demonstrate any causal effect. Therefore, at this point, the many statements along the manuscript inferring causality are misleading (for example title of Fig. 4, statement in lines 345-346, 379-380, 410-411, 436-437, 476, 480-482, 485).

Author response
We thank the reviewer for the valuable suggestions. We have rephrased the statements and incorporated necessary the changes in the revised manuscript.

Comment 9:

To investigate if rescue depends on mt dynamics, the authors should simultaneous eliminate Fzo1 and Dnm1, which allows to maintain mitochondrial tubulation and respiration. They should compare respiration, mitochondrial mass, cell cycle arrest and ROS levels of ubp2 hsp31 hsp32 cells and of fzo1 dnm1 ubp2 hsp31 hsp32 cells.
Of note: ubp2 cells have fragmented mitochondria, but do not affect mitochondrial mass, cell cycle progression, ROS levels or growth resistance to H2O2. This suggests that mitochondrial alterations per se do not cause cell cycle, ROS or mitochondrial mass defects.

Author response
Thank you for the valuable suggestion. We attempted constructing the strains as mentioned above but could not generate viable colonies of *Δ31Δ34Δubp2Δfzo1Δdnm1,* possibly due to the deletion of 5 genes together. However, the Fzo1 and Dnm1 levels were measured by Western blotting as indicated in **Fig. 4A** and **Fig. S12** respectively. Further, to substantiate the role of Ubp2 in the stabilization of the hyperfused mitochondrial structure, we overexpressed Ubp2 in WT background and performed phenotypic assessment, microscopy and immunoblotting to probe for Fzo1 levels (**Fig. S5**). The results further highlight the role of mitochondrial dynamics maintained by Ubp2.

Indeed, fragmentation of mitochondria in the absence of Ubp2 does not cause cell cycle, ROS, or mitochondrial mass defects in the conditions used in the present study.

Other points:

Comment 1:

6) To my knowledge, a causal effect of mitochondrial fusion and fission in cell cycle progression has not been demonstrated in S. cerevisiae, which compromises a few statements, e.g. lines 320-321. Please comment/correct.

Author response

Thank you for the suggestion. We have incorporated references indicating the role of mitochondrial dynamics in cell cycle progression in the revised version of manuscript.

Comment 2:

5) In their redox biol manuscript (PMID: 32070881), the authors show that hsp31 hsp32 cells have growth defects on glycerol similar to hsp31 hsp34. However, only the last one has increased mitochondrial mass and post-diauxic-shift-like mitochondria, while hsp31 hsp32 cells have wild-type like mitochondria. Therefore, the different phenotypes related to Hsp31 loss are not necessarily related. Please comment in the manuscript.

Author response

Thank you for the comment. Even though Δ*31*Δ*32* exhibits compromised growth phenotype, the mitochondrial morphology is not perturbed. It is possible that the growth phenotype associated with 32 paralogs may be due to other uncharacterized functions.

The Δ31Δ34, however, exhibits compromised phenotype due to increased mitochondrial function, suggesting that Hsp34 particularly sensitizes the mitochondrial functions performed by Hsp31 **Bankapalli, Kondalarao *et a*l. ,2020** (PMID:**32070881)**. Besides, the Hsp34 is much more divergent in the sequence in comparison with the Hsp32 and Hsp33 paralogs. Therefore, we believe that Hsp34 has better evolved vital functions in conjunction with Hsp31 in maintaining overall cellular and organellar health.

Comment 3:

6) In their mitophagy assessments, it would be advisable to perform FM464 co staining with OM45-GFP to demonstrate interiorization in to the vacuole.

Author response

Thank you for the suggestion. We have stained the vacuoles with FM4-64 and the new figure has been incorporated in **Fig. 3B**.

Comment 4:

7) It could be preferable to name WT-morphology as tubular, and hsp31 hsp34 as PDS-like.

Author response

Thank you for the suggestion. We have now incorporated the changes as suggested.

Comment 5:

8) The introduction could be shortened to the most relevant functions of Hsp31 and Ubp2 in context of this manuscript, given that no clinical aspects are addressed in the present study.

Author response

Thank you for the response. The introduction has now been shortened.
