# Supplementary material for: Ubp2 modulates DJ-1-mediated redox-dependent mitochondrial dynamics in Saccharomyces cerevisiae
Source: PLoS Genet. 2025 Jul 3;21(7):e1011353. doi: 10.1371/journal.pgen.1011353 (PMC12251144; doi:10.1371/journal.pgen.1011353)
Supplement: Attachment — Submitted filename: Author response_PGENETICS-D-2400709R2_100625.pdf [file pgen.1011353.s055.pdf]

Author response for Reviewers (after major revision)

Reviewer #1: I am satisfied that the authors have made the requested revisions to their manuscript apart from the following minor corrections:

Comment 2 - The author's response has clarified that Ubp2 expression is altered at the late log phase, as presented in Fig.1B and Fig.S1. However, this has not been clarified in the manuscript itself or legend for Figure 1 to state what growth phase was used. It is important to clarify this since the increase in Ubp2 expression is not observed at the same growth phase as that used for the spot tests.

Author response

We thank the reviewer for the critical assessment of the revised manuscript. We have now mentioned these changes in the figure legends (Fig. 1B) and the manuscript (refer to lines 192-198).

Comment 5 - The authors have added quantification for the mitophagy assay shown in Fig. 3 as a supplementary figure. This should be added as a panel to main Figure 3 along with appropriate statistics.

Author response

Thank you for the response. Fig. S4B is now Fig. 3B and appropriate statistics have been added.

Comment 8 – The authors have now included data describing the GSH:GSSG ration in mitochondria and cytosol. The Methods would benefit from more description of how these experiments were actually performed, particularly how mitochondrial and cytosolic extracts were prepared and verified

Author response

Thank you for the response. We have now included the method for isolation and separation of mitochondrial and cytosolic extracts in the section "Materials and Methods" (line 693-697).

<u>Reviewer #2:</u> **The authors have addressed all of the raised criticism in a point by point reply and have presented an improved manuscript.**

Minor issues which need adaptation from my point of view are limited to Fig. 7, where some pictures seem inapproprately contorted/ stretched and some labelling behind white boxes remains visible and Suppl. Fig. 3 where the white boxes do not fit to the 2x-zoom pictures. Also here it seems that some of the pictures are slightly contorted/stretched.

<u>Author response</u>

We thank the reviewer for the valuable inputs. We have incorporated the mentioned changes in the figures.

<u>Response to Reviewer 3 – Rebuttal After Appeal</u>

<u>Author's General Comment:</u>

We appreciate Reviewer 3's feedback and have performed experiments to clarify the rescue and causal effects, highlighting genetic interaction between Ubp2 and DJ-1 paralogs. We acknowledge that most phenotypes were presented after plasmid re-expression, and the data shown sufficiently supports the key claims. Below, we respond point-by-point to the reviewer's concerns, highlighting clarifications, additional data, and relevant context. **Reviewer 3 comments** for the revised version of the manuscript is highlighted in **red** and Reviewer 3 comments after initial submission have also been included. **Author's response after the revision** is highlighted in **blue** and **<u>author comments for the rebuttal are boxed</u>**.

<u>Reviewer 3 General comment (after major revision):</u>

"The revised version has only very minimal improvements, because the large majority of the requests were not performed. Globally, the rescue experiments

are either not presented or not convincing, and causality experiments have not been added."

Author response (rebuttal):

We respectfully clarify that plasmid re-expression experiments were performed for **most of the key phenotypes**, including those assessing the function of a **catalytically inactive cysteine mutant of Ubp2**. These experiments were **strategically selected** to address the core mechanistic questions posed in the study directly and to validate our central conclusions. While it is unjustifiable to repeat **the entire manuscript with plasmid re-expression**, the presented data represent a targeted and robust approach to demonstrating both rescue and causality. The results are consistent and reproducible, and we believe they sufficiently support the manuscript's main claims.

To clarify and summarize our findings:

- **Δ*31*Δ*34* exhibits hyperfused mitochondrial structures (Fig. 2) due to increased expression of Ubp2 (Fig.1), which stabilizes Fzo1 and promotes fusion (Fig. S4).**

- **Overexpression of Ubp2 in the Δ31Δ34Δubp2 background results in stabilization of Fzo1 (Fig. S5)**, which in turn **restores mitochondrial hyperfusion**, as reflected in the improved mitochondrial morphology and reduced fragmentation (Fig. S9 & S10).

- **The use of a catalytically inactive Ubp2 mutant failed to rescue these phenotypes (Fig. S8 to Fig. S12)**, reinforcing the importance of Ubp2's enzymatic activity in this pathway.

- **Moreover, loss of DJ-1 paralogs leads to elevated GSSG levels (Fig. 7B, C and Fig. S8)**, thereby mediating **increased Fzo1-dependent mitochondrial fusion**, suggesting that redox imbalance modulates this process.

These observations collectively support a **causal role for Ubp2 and DJ-1 paralogs in regulating mitochondrial morphology through redox-sensitive mechanisms and protein homeostasis.** We are providing a **graphical abstract** for a better understanding.

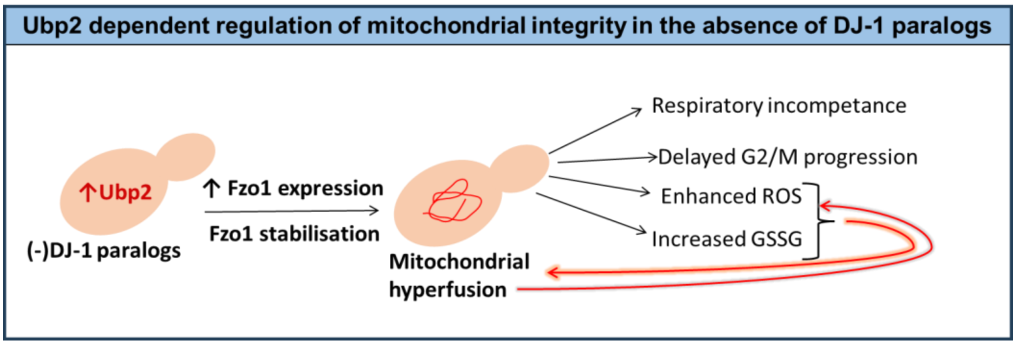

Reviewer 3 Comment 1A (after initial submission):

"They should provide evidence that re-expression of Ubp2 rescues all □hsp31□hsp34 phenotypes presented in this manuscript."

Author response (Revision):

Thank you for the valuable suggestions. We have re-expressed Ubp2 in the triple deletion strain (Δ*31*Δ*34*Δ*ubp2*) and analysed for the mitochondrial respiration competency **(Fig. 1D)**. Besides, the immunoblotting and cycloheximide chase assay also suggest stabilized expression of Fzo1 upon overexpression of Ubp2 **(Fig. S6).** Further, phenotypic analysis was performed to check for the response of cells to the treatment of H2O2 upon Ubp2 overexpression **(Fig. S7A).** DCFDA staining was also performed, and the microscopic analysis corroborated the observed phenotype **(Fig. S7B).**

Reviewer 3 Comment (after revision)

"*From these 8 phenotypes, the authors only attempt to address rescue for 4 phenotypes. Moreover, none of the 4 rescue experiments presented are convincing, as described below. Finally, overexpression is not appropriate: instead rescue experiments must be designed so that re-expression re-establishes WT-like levels. Yeast has a very big toolbox to allow it. Generally, this is achieved by centromeric plasmids. In addition, endogenous WT levels could be assessed by genomic HA tagging.*

The phenotypes presented are:

1)Respiratory competence, assessed by glycerol growth (Fig 1),

2)mt morphology (Fig. 2A,B),

3) mt mass (Fig. 2C), functional mt mass (Fig. 2D), and ATP levels (Fig. 2E)

4) mitophagy (Fig. 3A and B)

5) Fzo1 ST levels (Fig. 4A,B), stability (Fig. 4C,D), Fzo1 ubi (Fig. 4E,F),

6) cell cycle defect (Fig. 5),

7) ROS levels (Fig. 6A, B), and mt-ROS levels (Fig. 6C,D),

8) Ox stress resistance, by spot tests upon H2O2 (Fig 7A), and Glutathione levels (Fig. 7B,C)."

Author response (rebuttal):

We thank the reviewer for this detailed feedback. Due to the scope of the manuscript and technical limitations, **it was unjustifiable to repeat every phenotype with plasmid-based re-expression, thereby entailing repetition of the entire manuscript**. However, we have rescue experiments **for six of the eight phenotypes** in the manuscript. These include:

1. **Respiratory competence**, assessed by glycerol growth (**Fig. 1D and Supplementary Fig. S16**)
2. **Mitochondrial morphology** (**Fig. S12**)
3. **Mitochondrial mass (Fig. S13A), Functional mitochondrial mass (Fig. S13B)**
4. **Fzo1 steady state levels (Fig. S6A) and stability (Fig. S6B and Fig. S6C)**
5. **ROS levels (Fig. S7) and mt-ROS levels (Fig. S8)**
6. **Oxidative stress stress resistance, by spot tests upon H_2O_2 (Fig.S7 and Fig.S10), and Glutathione levels (Fig. S11)**

These phenotypes were strategically selected for rescue studies because they represent core cellular processes—**respiration, redox homeostasis, mitochondrial structure**, and **stress resistance**—most directly affected in the Δ*31*Δ*34* background.

We appreciate the reviewer's emphasis on the importance of physiologically relevant expression levels in rescue experiments. Indeed, yeast offers a robust toolkit—including centromeric plasmids and genomic HA tagging—to re-establish wild-type (WT)-like expression. The basal expression of Ubp2 has also been measured in both WT and Δ*31*Δ*34* by genomic tagging of Ubp2 with an HA tag in **Fig. 1B** and **Fig. S1B**. Consequently, Δ*31*Δ*34* cells exhibit a ~2-fold increase in Ubp2 levels upon deletion of Hsp31 paralogs, as shown in

our data. This elevated expression is not an artifact but a biologically relevant feature of the Δ*31*Δ*34* background.

Accordingly, using centromeric plasmids to reintroduce Ubp2 at WT levels would not accurately model the endogenous Ubp2 context in Δ*31*Δ*34* cells. We aimed to investigate phenotypic reversion under conditions that efficiently recapitulate the elevated Ubp2 levels observed in the Δ*31*Δ*34* background. Therefore, a higher level of Ubp2 expression was deliberately employed to reflect the physiological upregulation in this background.

Nevertheless, to rule out overexpression artifacts, we also expressed Ubp2 under its native promoter. We observed no change in Fzo1 levels (see blot attached in response to comment 1C), consistent with the results presented in the manuscript.

Reviewer 3 Comment (after revision)

(Referring to Fig. 1D)

"Not answered at this stage, because Fig 1D lacks the control D31D34 EV (the controls must be on the same plate, so this cannot be replaced by Fig. 1A).

From this point on D31D34 will be named DD and D31D34Dubp2 will be named DDD"

Author response (rebuttal):

We would like to clarify the experimental design presented in Fig. 1D. It has been consistently shown throughout the manuscript that the Δ*31*Δ*34*Δ*ubp2* strain closely resembles WT, both in terms of respiratory function and mitochondrial dynamics, reinforcing the role of Ubp2 as a genetic interactor of DJ-1 paralogs.

In Fig. 1D, we chose Δ*ubp2* + Ubp2 (↑) as the control strain for plasmid-based rescue to demonstrate that:

- Overexpression of Ubp2 fully rescues the Δ*ubp2* phenotype, and
- Ubp2 overexpression is not intrinsically toxic in cells lacking Ubp2.

Δ*31*Δ*34* strain was omitted from the spot assay based on the understanding that its phenotype was already well-established and unchallenged in the initial

round of reviews. However, following the rejection of the revised version**, we have incorporated a spot assay with Δ*31*Δ*34*/EV along with the rest of the strains on one plate. Please refer to Fig. S13. Consistent with the results, Δ31Δ34Δubp2/ Ubp2 (↑) exhibits respiratory incompetence comparable to Δ31Δ34 /EV.**

Reviewer 3 Comment (after revision)

(Referring to Fig. S6)

"Not convincing at this stage, lacks quantifications and statistics. How do the authors explain an increase in Fzo1 levels in the CHX chase, despite a rather even loading control?"

Author response(rebuttal):

We thank the reviewer for this valuable observation. In response, we have **updated Fig. S6** to include the **quantification of Fzo1 band intensities** and the **statistical analysis across biological replicates**, now clearly shown alongside the blot (Fig.S6).

Reviewer 3 Comment (after revision)

"The image S7A is convincing. How many times was it reproduced?"

Author response (rebuttal):

We thank the reviewer for their positive feedback on **Fig. S7A**. The experiment shown was **reproduced independently three times, each yielding consistent results**. Representative images from these biological replicates were assessed for reproducibility, and the version included in the manuscript reflects the typical phenotype observed across all trials. Also, please refer to **Fig. S10**, which shows a phenotype similar to Fig. S7A upon Ubp2 overexpression. We can provide additional replicate images in the supplementary data if required.

Reviewer 3 Comment (after revision)

(Referring to Fig. S7B)

"Not convincing, due to lack of controls (as a minimum DD EV is necessary), lack of quantification and lack of statistics. Besides, please comment on the fact that DDD+Ubp2 from Sup 7B does not resemble at all the DD in Fig. 7B/C. In fact, in Fig. 7B (and quantification in 7C) the DD has less staining for Glutathione than WT or Dubp2, and the DDD resembles WT. Therefore, in case of a rescue by re-expression of Ubp2, DDD+Ubp2 should have less staining than WT. INSTEAD, Fig. S7B shows that DDD+Ubp2 has even much higher values than the DDD EV (moreover, in S7B DDD EV has higher signal than WT EV, IN CONTRAST to Fig. 7C)"

Author response (rebuttal):

The data for quantifications in all the figures do not indicate the intensity calculation of microscopy. Rather, the microscopy results were validated with flow cytometry as another technique. We have provided the **flow cytometry data for DCFDA staining (Fig. S7C) and used Δ*31*Δ*34*/EV in the panel (Fig. S7B and Fig. S7C).** Further**,** we want to clarify the purpose and interpretation of **Fig. S7B** and **Fig. 7B/C**:

1. Fig. S7B utilizes DCFDA staining, which measures total intracellular ROS levels.

2. Fig. 7B/C employs monochlorobimane (MCB) staining, selectively detecting intracellular reduced glutathione (GSH). Lower fluorescence intensity corresponds to reduced GSH availability, indicating higher oxidative stress.

Therefore, **upon overexpression of Ubp2 in Δ*31*Δ*34*Δ*ubp2* (see panel 3 in Fig. S7B), there is increased DCFDA staining compared to WT. Consistently, overexpression of Ubp2 in Δ*31*Δ*34*Δ*ubp2* has lesser MCB staining (see panel 3 in Fig. S11), indicating lower glutathione levels and increased ROS.** In both S7B and S11, DCFDA and MCB staining of Δ31Δ34Δubp2/Ubp2(↑) are comparable to Δ31Δ34/EV, respectively. Therefore, Δ31Δ34Δubp2/Ubp2(↑) in S11 indicates lower GSH levels(by

MCB), while Δ31Δ34/EV in Fig.S7B/C indicates higher ROS (by DCFDA) correlating with each other.

Considering Fig. S7B, indeed, **Δ31Δ34Δubp2/Ubp2↑ has higher DCFDA staining than Δ31Δ34Δubp2/EV**, in line with the depicted results. This observation reinforces our model that precise regulation of Ubp2 levels is critical, and its overexpression in a DJ-1 paralog-deficient background exacerbates oxidative damage.We hope this distinction clarifies the datasets and highlights the complementary nature of the assays used.

Reviewer 3 Comment (after revision)

Referring to Fig. S8 (Now S12)

“Not convincing. Lacks convincing images (I fail to see clear differences between the 4 panels) and lacks quantification.

In sum, this point 1A is NOT convincingly answered. Without the rescue the phenotypes cannot be validated.”

Author response (rebuttal):

In Fig. S8 (Now **Fig. S12**), we analyzed the **mitochondrial network morphology upon Ubp2 overexpression in *Δ31Δ34Δubp2*** and report that the tubular structures are reinstated in *Δ31Δ34Δubp2*/Ubp2↑ strain **(see panel 5 in Fig.S12**). Quantifications have been added for the depicted mitochondrial morphologies **(Fig.S12)**.

We want to emphasize that a **substantial portion of the manuscript has been re-evaluated and validated using plasmid-based re-expression of Ubp2**. These experiments were carefully designed and executed to address the reviewer’s concerns, and the resulting data align with and reinforce the conclusions drawn from the original experiments. The consistency between the two approaches strengthens our findings' validity and supports the robustness of our conclusions. These re-expression studies addressed key mechanistic aspects and reinforced Ubp2 as the player that cross-talks with DJ-1 paralogs in maintaining redox homeostasis.

Reviewer 3 Comment 1B (after the initial submission):

B) Moreover, is this rescue dependent on the catalytic activity of Ubp2?

Author response(revision):

We thank the reviewer for the valuable suggestion. A catalytically dead mutant of Ubp2 was constructed by mutating Cysteine to Serine mutation at the 745 residues (**Lam, Mandy H Y et al.** PMID:**19165343**), (**Santos, Clara M *et al*.PMID: 39384040**). We visualized the mitochondrial network morphology upon overexpression of Ubp2 C745S in Δ*31*Δ*34*Δ*ubp2*, and the mitochondrial structures resembled WT **(Fig. S8, panel 4).** This suggests that the catalytic cysteine is indispensable for maintaining the function of mitochondrial fusion.

Reviewer 3 Comment 1B (after revision):

"Not convincing, as described above. Data for all other 7 phenotypes is lacking."

Author response (rebuttal):

Ubp2 is reported to be a cysteine-dependent deubiquitinase, with previous studies confirming that cysteine mutants (such as C745S) are catalytically inactive (Lam et al., 2009, PMID: 19165343; Santos et al., 2022, PMID: 39384040). In order to assess the necessity of Ubp2's catalytic cysteine in mediating the phenotypes observed in this study, we constructed a **catalytically dead Ubp2 mutant (C745S) to specifically analyze the role of the catalytic cysteine**.

Repeating the entire experimental workflow with the Ubp2 mutant would result in redundancy and is scientifically unwarranted, given that the objective was to assess the contribution of Ubp2's catalytic activity specifically in the context of the phenotypes observed in cells lacking Hsp31 paralogs. Therefore, we focused on evaluating **key phenotypes** following re-expression of the catalytically inactive **Ubp2 mutant (C745S)**. Moreover, it is scientifically unjustified to invalidate the study on the grounds of not repeating every experiment with the catalytically inactive mutant. The phenotypic validation provided here offers sufficient evidence that the observed effects depend on the catalytic activity of Ubp2, supporting the conclusions drawn in this study.

These phenotypes were chosen based on their **relevance to the core findings** of the study and include:

1. Analysis of mitochondrial network morphology (**Fig. S12, panel 6**)
2. Assessment of basal mitochondrial ROS by MitoSox (**Fig. S8**)
3. Assessment of basal cellular ROS by DCFDA (**Fig. S9**)
4. Phenotypic assessment with Ubp2 mutant after H_2O_2 stress (**Fig. S10**)
5. Estimation of glutathione using monochlorobiamine (**Fig. S11**)

These experiments were specifically designed to understand the role of the **catalytic cysteine of Ubp2 in regulating mitochondrial morphology, ROS parameters, and sensitivity to extraneous stressors, the key phenotypes that are altered in cells lacking DJ-1 paralogs.**

Reviewer 3 Comment 1C (after the initial submission):

C) If yes, how do the authors explain that UBP2 deletion rescues □hsp31□hsp32 phenotypes, given that Ubp2 is expected to have reduced activity in □hsp31□hsp32 cells. This prediction is because □hsp31□hsp32 cells have increased ROS levels (PMID: 37548361and 32070881) and oxidative stress inactivates the catalytic activity of Ubp2 (PMID: 25622294).

Author response(revision):

Thank you for the response. Yes, the catalytic activity of Ubp2 is necessary for mediating the rescue observed in *Δ31Δ34Δubp2*. As reported previously, Ubp2 is inactivated reversibly in response to H2O2 stress (0.6mM- 2.5mM) (**Silva, Gustavo M et al.**PMID: **25622294**) as an extraneous stressor. However, in the absence of DJ-1 paralogs, there is an inherent increase in the ROS levels, maybe less than what we used for the experimental condition that we genuinely believe. At this, lower ROS levels in the absence of DJ-1 paralogs plausibly do not inactivate the catalytic cysteine of Ubp2. Moreover, Ubp2 is expressed more in *Δ31Δ34* than WT **(Fig 1B),** leading to higher activity causing hyperfusion of mitochondrial structures without DJ-1 paralogs.

Reviewer 3 Comment 1C (after revision):

“Although requested, this was not shown, as commented above. At this stage, this statement is a hypothesis. It should be validated experimentally.

Meaning: Ubp2 is ca 2X more abundant in DD than in WT. Does mild overexpression of Ubp2 (ca 2X) in WT cells resemble DD for any of the 8 phenotypes presented?

Of note: the mt morphology results presented in Fig. S5B and commented bellow, at the end of point 5, does not support the author’s statement. DD resemble PDS phase cells, and this does not appear to be the case for WT+Ubp2.”

Author response Comment 1C (rebuttal):

We want to highlight that this study emphasizes the role of Ubp2 in regulating mitochondrial health and redox imbalance upon combinatorial deletion of Hsp31 and Hsp34 (not Hsp31 and Hsp32, as has been mentioned in the trailing comments by Reviewer 3). **Please read “□hsp31□hsp32” in Reviewer 3 comments as Δ*31*Δ*34***. Our results suggest that the catalytic cysteine of Ubp2 is essential for regulating mitochondrial fusion, but there is **no evidence of lower activity of Ubp2 when the “basal” ROS levels are high in the present manuscript**. Hence, assuming that Ubp2 is functionally repressed in the Δ*31*Δ*34* background would be incorrect. On the contrary, our data indicate that Ubp2 expression is upregulated—approximately two-fold—in the absence of Hsp31 paralogs, and functional assays, including phenotypic reversion and Fzo1 level and ubiquitination analysis, support the conclusion that Ubp2 remains active under these conditions. **The current manuscript aims to elucidate the mechanism underlying mitochondrial hyperfusion, specifically in DJ-1 paralog deletion.** Through genetic and biochemical analyses, we identify **Ubp2 as a key genetic interactor of Hsp31 and Hsp34**, which together regulate mitochondrial dynamics. Our findings demonstrate that **Ubp2 mediates mitochondrial hyperfusion in cells lacking both Hsp31 and Hsp34**, highlighting a novel functional axis between redox regulation and mitochondrial morphology.

However, Western blotting for Fzo1 ubiquitination after pull-down was designed particularly as a read-out of Ubp2 expression and activity, as Fzo1 is a known substrate of Ubp2 (PMID: 28607491)

Ubiquitination status of Fzo1 after pull down in Fig. 4E &F suggests that:

1. Δ*ubp2* exhibits enhanced ubiquitination of Fzo1, indicating faster degradation of Fzo1(**Fig. 4C&D**) and presence of fragmented mitochondria (**Fig. 2A&B**) in comparison to WT.
2. **Δ*31*Δ*34* exhibits reduced ubiquitination of Fzo1, correlating with slower degradation of Fzo1(Fig. 4C&D)** and presence of hyperfused mitochondria (Fig. 2A&B) in comparison to WT.

These results suggest that Ubp2 activity is not abrogated in Δ*31*Δ*34*. Instead, due to the increased expression of Ubp2, its deubiquitination activity on Fzo1 is enhanced, thereby promoting hyperfusion in the absence of DJ-1 paralogs. Enhanced expression and, thereby, the activity of Ubp2 promote Fzo1 fusion. As previously reported, Ubp2 is **reversibly inactivated** in response to **extrinsic oxidative stress induced by H_2O_2 (0.6 mM–2.5 mM)** (Silva, Gustavo M. et al., PMID: 25622294). In contrast, in the absence of DJ-1 paralogs, there is an inherent elevation in cellular ROS levels, although likely lower than the millimolar concentrations of H_2O_2 treatments provided extrinsically in PMID: 25622294. We believe that these moderately **increased basal ROS levels in Δ*31*Δ*34* are insufficient to inactivate the catalytic cysteine of Ubp2, thereby allowing its continued activity under basal oxidative conditions, in line with the study's results.**

We appreciate the reviewer's thoughtful mechanistic consideration. However, we would like to clarify that the manuscript does not make any claims regarding the enzymatic activity of Ubp2 in the Δ*31*Δ*34* background. Our study focuses on the genetic interaction between Ubp2 and the DJ-1 paralogs and the resulting phenotypic consequences rather than the enzymatic status of Ubp2 under basal oxidative conditions. Therefore, the conclusions are based on functional outcomes, not inferred enzymatic activity changes.

The experiments were designed in alignment with the results we observed regarding the use of centromeric plasmids. Specifically, deletion of the Hsp31 paralogs leads to an approximately two-fold increase in Ubp2

expression, an essential factor we considered when choosing expression systems. Given this, using plasmids that merely restore WT-level expression would not appropriately model the elevated Ubp2 context observed in the Δ*31*Δ*34* background. To investigate phenotypic reversion accurately, higher expression of Ubp2 is required; hence, mild centromeric plasmids would not suffice for this purpose. **Yet, we probed for Fzo1 levels after expressing Ubp2 in its native promoter and found no difference in Fzo1 levels, consistent with the results in the manuscript. We have attached an image of the blot in the rebuttal letter.**

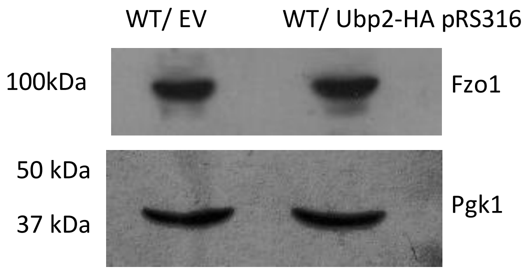

<u>Reviewer 3 Comment 1D (after the initial submission):</u>

D) Along this line, repressed activity of Ubp2 is expected to cause mitochondrial fragmentation, but the □hsp31□hsp32 cells have increased mitochondrial tubulation. How do the authors explain this?

Author response

Thank you for the critical response. Ubp2 is expressed more in the absence of DJ-1 paralogs and is plausibly not inactivated by intrinsic ROS. Thus, enhanced expression of Ubp2 stabilizes Fzo1 and causes increased mitochondrial tubulation.

<u>Reviewer 3 Comment 1D (after revision):</u>

"This assumption requires experimental validation."

<u>Reviewer 3 Comment 1D (rebuttal):</u>

The current manuscript provides no evidence of Ubp2's repressed activity in the absence of DJ-1 paralogs**. Contrarily, the ubiquitination status of Fzo1 in Δ*31*Δ*34* suggests the presence of enhanced Ubp2 activity due to its enhanced expression. Hence, we observe increased mitochondrial tubulation in the absence of DJ-1 paralogs.**

Notably, the catalytically inactive mutant of Ubp2 was unable to induce mitochondrial hyperfusion and the associated redox imbalance, even upon overexpression **(Fig. S8 to Fig. S12). This observation reinforces the idea that the intrinsic ROS generated in the absence of Hsp31 paralogs is insufficient to inhibit Ubp2 activity.** Consistent with previous findings, Ubp2 requires relatively high levels of oxidative stress—specifically, H_2O_2 in the millimolar range—for reversible inactivation of its catalytic cysteine (Silva, Gustavo M. et al., PMID: 25622294). Therefore, the basal ROS levels present in the absence of DJ-1/Hsp31 paralogs likely fall below this threshold, allowing Ubp2 to remain functionally active.

<u>Reviewer 3 Comment 2 (after the initial submission):</u>

2) Related to point 1), can it be that catabolic repression caused by growth in glucose is absent in □hsp31□hsp32 cells? Consistently, mitochondrial morphology of the □hsp31□hsp32 cells intriguingly resembles WT cells in post-diauxic shift phase (PDS). Therefore, the authors should test if PDS cells resemble □hsp31□hsp32 cells in cell cycle progression, mitophagy, ROS levels or growth resistance to H2O2. If yes, are these effects in PDS cells rescued by deletion of UBP2?

Author response

Thank you for your critical response. The cells in post-diauxic shift were phenotypically assessed in the presence of H2O2. However, the PDS cells exhibit resistance to oxidative stress **(Fig. S9)**. Cell cycle progression and mitophagy assessment could not be performed due to the technical drawbacks in designing the experiments. For instance, in the current study, the cells were arrested in the G1 phase to synchronize the culture. However, synchronization of cells in PDS could not be performed as PDS cells were not responsive to the alpha factor. Similarly, for mitophagy induction, cells in the mid-log phase are transferred from dextrose-containing media to glycerol, and samples are collected over the indicated time points. Hence, cells in post-diauxic shift could not be analyzed for mitophagy. From the experiment performed, even though *Δ31Δ34* resembles the mitochondrial morphology of PDS cells, the PDS cells are oxidative stress resistant. Previous reports also

indicate stress resistance to hydroperoxides in cells undergoing post-diauxic shift (**Maris, A F et al.** PMID: **11409175).**

Reviewer 3 Comment 2 (after revision)

Referring to Fig. S18 (previously S9)

"The author's answer is satisfactory."

Author response to Comment 2 (rebuttal)

We thank you for the positive response, but we want to clarify that Δ*31*Δ*34* does not phenocopy the cells in the post-diauxic shift phase (PDS phase cells) as observed in **Fig. S18 (previously Fig. S9)**. Therefore, based on the above evidence, it would be inaccurate to conclude that Δ*31*Δ*34* cells resemble PDS phase cells.

Reviewer 3 Comment 3 (after the initial submission):

3) Related to point 2) is the rescue of respiration, mitochondrial mass, cell cycle arrest and ROS levels observed in □ubp2□hsp31□hsp32 cells rather due to effects in the TCA and ETC pathways? The authors could follow an approach similar to https://doi.org/10.1101/2024.07.09.602707. For example, is the rescue dependent on the carbon source? What happens upon deletion of RTG1 or Hap4 overexpression?

Reviewer 3 Comment 2 (after revision)

Referring to Fig. S19 (previously S10)

"The author's answer is satisfactory."

Author comment (rebuttal)

Please note that Fig. S10 is now Fig. S19.

Reviewer 3 Comment 3 (after the initial submission):

4) □hsp31□hsp32 cells have altered levels and stability of Fzo1. How do these chaperones affect Fzo1? The authors should test if this is caused by an increase in mitochondrial biogenesis.

Of note, in PMID: 32070881 they observe that □hsp31□hsp32 cells have increased transcriptional of all 3 major mt dynamics proteins: Dnm1, Mgm1 and Fzo1, and very comparable increases in their respective protein levels. Consistently, in this same manuscript, they show that □hsp31□hsp32 cells

have increased mitochondrial mass and increased GSSG levels, thus altering the GSH/GSSG ratio. Reduction in GSH is an indicator of oxidative stress and they could revert increased mitochondrial mass with the antioxidant NAC.

Author response (revision):

We thank the reviewer for the suggestion.Indeed, hyperfusion caused due to stabilized Fzo1 also causes increased biogenesis as indicated by increment in expression of mitochondrial proteins (Tom70,Tim44, Tim23) as mentioned in **Bankapalli, Kondalarao *et al.* ,2020** (PMID:**32070881**). The GSH and GSSG levels have been represented as ratios in the mitochondrial and cytoplasmic extracts. Kindly refer to **Fig. 7D and Fig. 7E** in the revised manuscript.

Reviewer 3 Comment 2 (after revision)

"*The request in this point was to revert the increase in mt mass they observe in DD (e.g. by adding NAC to DD, as in PMID: 32070881) and then check Fzo1 levels and mt morphology.*

Regarding the answer given by the authors, once again it should be noted that a correlation (simultaneous observation of increased Fzo1 and increased Tom and Tim components) does not mean causality."

Author response (rebuttal)

Mitochondrial biogenesis refers to generating new mitochondrial mass, including upregulating key mitochondrial proteins such as components of the TIM/TOM complexes (PMCID: PMC3883043; PMCID: PMC8926401). Our previous work (PMID: 32070881) demonstrated that DJ-1 paralogs regulate mitochondrial biogenesis and outlined a mechanistic model in which oxidized glutathione (GSSG) promotes Fzo1-mediated mitochondrial fusion. Given this established link, we found Reviewer 3's comment somewhat puzzling, as our earlier study described these regulatory mechanisms in detail.

Nonetheless, in response to the reviewer's suggestion in the revised manuscript, we conducted additional experiments to strengthen our findings. **We treated Δ*31*Δ*34* cells with the antioxidant N-acetyl cysteine (NAC) and assessed three key mitochondrial parameters: Fzo1 levels (Fig.**

S17B), mitochondrial morphology (Fig. S17A), and total mitochondrial mass (Fig. S17C). Consistent with our hypothesis, NAC treatment restored all three parameters, supporting the conclusion that redox imbalance resulting from the deletion of Hsp31 paralogs affects mitochondrial function by modulating Fzo1 levels.

<u>Reviewer 3 Comment 5 (after the initial submission):</u>

5) They infer that UBP2 deletion rescues the cellular defects of □hsp31□hsp32 cells due to the ubiquitination, stability and steady state levels of Fzo1. However, besides presenting correlative data, they do not demonstrate any causal effect. Therefore, at this point, the many statements along the manuscript inferring causality are misleading (for example title of Fig. 4, statement in lines 345-346, 379-380, 410-411, 436-437, 476, 480-482, 485).

<u>Reviewer 3 Comment 5 (after revision)</u>

"Can it be that the authors did not submit the correct manuscript text? Because except in one case, no alterations were made.

The only statement altered, in lines 363-366 (previously lines 345-346) "Taken together, the results indicate that remission of mitochondrial structures upon deletion of Ubp2 in the absence of Hsp31 paralogs enables the cells to undergo unimpeded progression through the cell cycle.": IS STILL MISLEADING. Replacement of "mt dynamics" by "mt structure" doesn't solve the lack of causality.

All other statements were not altered. Therefore, as before, statements in lines 409-410 (previously lines 379-380), in lines 445-446 (previously 410-411), in lines 480-481 (previously 436-437), in line 525 (previously 476), in lines 529-532 (previously 480-482), and in lines 534-535 (previously 485) ARE NOT CORRECT, because CAUSALITY WAS NOT DEMONSTRATED FOR ANY OF THESE STATEMENTS. The manuscript only presents correlations."

Author response (rebuttal)

We want to clarify that the claims made in the manuscript are strictly based on the experimental results and do not involve any exaggeration of the data presented. The conclusions have been drawn directly from observed phenotypes and validated through appropriate assays. These are not merely correlational but are supported by functional analyses and genetic interactions. Based on the line numbers from the **revised manuscript** (as there is a discrepancy in stating line numbers by Reviewer 3), we have highlighted comments deemed "misleading" as follows:

1. Title of Fig. 4: **Ubp2 deletion restores cell cycle progression in cells lacking Hsp31 paralogs (Now lines 354-355)**

Author response (rebuttal)

In Fig. 5A, the cell cycle analysis reveals that *Δ31Δ34* cells exhibit delayed progression compared to wild-type (WT), indicating a cell cycle defect. In contrast, *Δubp2* cells show progression comparable to WT. Notably, the triple mutant *Δ31Δ34Δubp2* restores cell cycle kinetics, resembling WT. These findings support the conclusion that **deletion of Ubp2 rescues the cell cycle delay explicitly observed in the absence of Hsp31 paralogs**, consistent with the statement in the manuscript.

.line 363-366 (Now lines 371-375) and line 525 (Now 547): Interestingly, restoration of mitochondrial integrity in Δ*31Δ34Δubp2* led to the usual progression in cell cycle and is comparable to the WT. Taken together, the results indicate that remission of mitochondrial structures upon deletion of Ubp2 in the absence of Hsp31 paralogs enables the cells to undergo unimpeded progression through the cell cycle.

Author response (rebuttal)

Mitochondrial distribution is a prerequisite for unimpeded cell division and proper cell cycle progression. Hyperfused mitochondrial networks have been reported to cause an accumulation of cells in the G2/M phase, thereby delaying cell cycle progression (PMID: 24632851). In the present study, *Δ31Δ34* cells exhibit hyperfused mitochondria and a corresponding

delay in cell cycle progression, consistent with previous findings, with a noticeable accumulation of cells in the G2/M phase. However, deletion of *Ubp2* in this background restores normal mitochondrial morphology and function. As a result, cell cycle progression is normalized in *Δ31Δ34Δubp2* cells, likely due to the re-establishment of mitochondrial parameters, which facilitates effective redistribution of mitochondria during cell division.

3. lines 409-410 (now 426-427): Ubp2 modulates oxidative stress sensitivity in GSH dependent manner, AND lines 379-380 (Now 463-465): As mitochondrial parameters of *Δ31Δ34* are altered in a redox-dependent manner by perturbation of the fusion protein, the GSH pool is also altered significantly

Author response (rebuttal)

Deletion of Ubp2 restores the glutathione levels by reinstating the mitochondrial activity (Fig. 1 and Fig. 2) in Δ*31*Δ*34*Δ*ubp2*, justifying lines 426-427. Further overexpression of Ubp2 in Δ*31*Δ*34*Δ*ubp2* reduces the GSH levels, highlighting the role of Ubp2 in regulating GSH-dependent redox balance in the absence of DJ-1 paralogs (Fig. S11).

The statement (in line 463-465) is omitted now for a more lucid understanding.

lines 480-481 (Now 502-504): The perturbed mitochondrial dynamics in the absence of Hsp31 paralogs is due to altered expression of mitochondrial fusion protein, particularly Fzo1

Author response (rebuttal)

As shown in Fig. 4, *Δ31Δ34* cells display increased expression and stabilization of Fzo1, implicating Fzo1 as a key driver of the hyperfused mitochondrial phenotype observed in the absence of Hsp31 paralogs. In contrast, the steady-state levels of Dnm1 remain unchanged across all strains (Fig. S15), reinforcing that the hyperfusion is primarily due to elevated Fzo1 and not diminished mitochondrial fission. These findings underscore Fzo1's central role in governing mitochondrial morphology in *Δ31Δ34*.

lines 529-532 (now in 550-552): Hsp31 paralogs are known to protect cells from carbonyl stress, thereby alleviating the concomitant formation of ROS species. Hence, cells lacking Hsp31 paralogs exhibit elevated levels of basal ROS [16]. This is mainly due to cells lacking Hsp31 paralogs exhibiting enhanced functional networked mitochondrial structures as an adaptive strategy, thereby increasing basal ROS levels

Author response (rebuttal)

Hsp31 paralogs regulate the redox status of cells by alleviating carbonyl stress (PMID: 37548361). **In the absence of these paralogs, elevated basal reactive oxygen species (ROS) levels—driven by an increase in oxidized glutathione (GSSG)—can shift the cellular redox environment.** Several studies have reported that **mitochondrial hyperfusion acts as an adaptive response to various stressors, including oxidative stress** (PMID: 19360003, PMID: 23861961, PMID: 34870686). This hyperfusion facilitates the exchange of mitochondrial contents such as DNA, proteins, and metabolites, thereby aiding in the repair of damaged mitochondria and preservation of mitochondrial function (PMID: 22936770). **Thus, the observed mitochondrial hyperfusion in Δ*31*Δ*34* cells likely reflects a stress-adaptive response to redox imbalance (PMID: 32070881).**

Lines 534-535 (now 556-558) Upon deletion of Ubp2 together with the Hsp31 paralogs suppresses the mitochondrial hyperfusion, thereby restoring the basal redox homeostasis

Author response (rebuttal)

In the Δ*31*Δ*34* background, elevated oxidative stress leads to mitochondrial hyperfusion—an adaptive response that preserves mitochondrial function under redox imbalance (PMID: 32070881). This hyperfused network maintains a higher **functional mitochondrial mass**, which sustains mitochondrial metabolic activity (indicated by increased ATP levels in Δ*31*Δ*34* in Fig. 2E) and contributes to elevated ROS levels. When **Ubp2 is deleted**, mitochondrial hyperfusion is suppressed due to increased ubiquitination and degradation of Fzo1, leading to a a reduction in total functional mitochondrial mass.

This **reduction in active mitochondrial mass (Fig. 1D)** decreases electron transport chain activity (indicated by lower ATP levels in Fig. 2E) and lowers ROS generation. Since mitochondria are the major source of intracellular ROS, **Ubp2 deletion limits oxidative stress by reducing mitochondrial activity**, thereby restoring **redox homeostasis**. Therefore, the deletion of Ubp2 restores mitochondrial morphology and **mitigates ROS accumulation** by reducing the overall oxidative burden associated with excessive mitochondrial fusion and function; thereby restoring basal redox homeostasis.

2. lines 433-435 (Now lines 450-452): Interestingly, the GSH and GSSG ratio is also a crucial indicator of mitochondrial fusion, whereby GSSG mediates the stability of mitofusins and promotes mitochondrial fusion in conditions of oxidative stress [45].

Author response (rebuttal)

The cellular redox environment tightly regulates mitochondrial fusion. Under oxidative stress, elevated levels of oxidized glutathione (GSSG) induce the oligomerization of mitofusin proteins, facilitating mitochondrial fusion (PMID: 22945481). We report that Δ*31*Δ*34* has elevated levels of GSSG, promoting mitochondrial fusion, in line with the study published by Bankapalli et.al (PMID: 32070881). Therefore, increased GSSG promotes hyperfusion in the absence of DJ-1 paralogs, as published in previous reports

Reviewer 3 Comment 5 (after the initial submission):

5) To investigate if rescue depends on mt dynamics, the authors should simultaneous eliminate Fzo1 and Dnm1, which allows to maintain mitochondrial tubulation and respiration. They should compare respiration, mitochondrial mass, cell cycle arrest and ROS levels of□□ubp2□hsp31□hsp32 cells and of □fzo1□dnm1□ubp2□hsp31□hsp32 cells.

Author response

Thank you for the valuable suggestion. We attempted constructing the strains as mentioned above but could not generate viable colonies ofΔ*31*Δ*34*Δ*ubp2*Δ*fzo1*Δ*dnm1,* possibly due to the deletion of 5 genes together.

However, the Fzo1 and Dnm1 levels were measured by Western blotting as indicated in **Fig. 4A** and **Fig. S12** respectively.

Further, to substantiate the role of Ubp2 in the stabilization of the hyperfused mitochondrial structure, we overexpressed Ubp2 in WT background and performed phenotypic assessment, microscopy and immunoblotting to probe for Fzo1 levels (**Fig. S5**). The results further highlight the role of mitochondrial dynamics maintained by Ubp2.

Reviewer 3 Comment 5 (after revision)

"This is a critical point for causality demonstration. Dnm1 dominant negative forms are available and could perhaps be integrated. Besides, S12 has no quantifications."

Author response (rebuttal)

We attempted to generate strains with simultaneous deletions of Dnm1 and Fzo1 along with the additional three gene deletions of Hsp31, Hsp34 and Ubp2; however, the **resulting strains were not viable**, likely due to the cumulative genetic burden. Moreover, such combined deletions would be confounding in the context of this study, which specifically aims to dissect the role of mitochondrial dynamic proteins. Introducing both fission and fusion defects would obscure the individual contributions of these pathways, thereby complicating the interpretation of our results.

Moreover, using a dominant-negative form of Dnm1 would necessitate a complete shift in the strain background, diverging from the one consistently used throughout this study. Such a change would require us to replicate the entire experimental framework in a different strain, introducing unnecessary variability and compromising the integrity and continuity of our findings. Given these constraints, this is technically beyond the scope and scientifically unwarranted. Quantification was not provided as the Dnm1 levels remain unaltered across the strains in **Fig. S12** but we have now incorporated the quantification in **Fig. S12** along with statistical analysis.

Reviewer 3 Comment 5 (after revision)

Referring to Fig. S5

“Differences are not convincing, and no quantification is presented. Besides, DD cells resemble PDS phase morphology, which is not the case in WT+Ubp2.”

Author response (rebuttal)

We have now included **quantifying cells with fragmented, intermediate, and tubular mitochondrial structures in Fig. S5,** and as mentioned before, referring to Δ*31*Δ*34* as PDS phase cells is incorrect. **The WT cells overexpressing Ubp2 exhibit tubular mitochondrial morphology, as depicted in Fig. S5**. We have even checked for respiratory competence and Fzo1 levels to validate the results **(Fig. S5)**. These results are in line with the previously stated data in the manuscript. The current data and supporting quantifications are now included to support our claims.

Reviewer 3 Comment 5 (after the initial submission)

Of note: ubp2 cells have fragmented mitochondria, but do not affect mitochondrial mass, cell cycle progression, ROS levels or growth resistance to H2O2. This suggests that mitochondrial alterations per se do not cause cell cycle, ROS or mitochondrial mass defects.

Author response

Indeed, fragmentation of mitochondria in the absence of Ubp2 does not cause cell cycle, ROS, or mitochondrial mass defects in the conditions used in the present study.

Reviewer 3 Comment 5 (after revision)

“As mentioned before, this suggests that mitochondrial alterations per se do not cause cell cycle, ROS or mitochondrial mass defects.”

Author response (rebuttal)

As explicitly stated in the manuscript and shown in **Fig. 2C,Fig. 5A,Fig.6 and Fig. 7,** mitochondrial fragmentation observed in the Δ*ubp2* strain does **not**

affect total mitochondrial mass, ROS response, or cell cycle progression **under the tested conditions**. **Rather, the reversal of phenotypes is specifically observed only when Ubp2 is deleted in the Δ*31*Δ*34* background, highlighting a genetic interaction between Hsp31 paralogs and Ubp2.** This specificity suggests that the phenotypic changes upon Ubp2 deletion, such as normalization of mitochondrial morphology, redox status, and cell cycle progression in **Δ*31*Δ*34*Δ*ubp2***, are **not global or nonspecific effects** of Ubp2 loss. Instead, they emerge **specifically in Hsp31 paralog deletion**, indicating a functional interplay between these proteins in regulating mitochondrial dynamics and cellular redox balance.

Reviewer 3's generalized statement extrapolates beyond what our data demonstrate. In this context, fragmentation is not associated with functional impairment of the aforementioned phenotypes in **Δ*ubp2***, and it is **misleading** to suggest that all mitochondrial alterations **do not carry physiological consequences at all conditions.**

<u>OTHER POINTS BY REVIEWER 3:</u>

<u>Reviewer 3 Comment 6 (after the initial submission)</u>

6) To my knowledge, a causal effect of mitochondrial fusion and fission in cell cycle progression has not been demonstrated in S. cerevisiae, which compromises a few statements, e.g. lines 320-321. Please comment/correct.

Author response

Thank you for the suggestion. We have incorporated references indicating the role of mitochondrial dynamics in cell cycle progression in the revised version of manuscript.

<u>Reviewer 3 Comment 5 (after revision)</u>

"Where exactly?"

<u>Author response (rebuttal)</u>

As cited in **lines 327–328 (references 42, 43, 44)**, mitochondrial structural transitions are integral for cells progressing through subsequent cell cycle phases. These studies underscore a **causal relationship between mitochondrial dynamics and cell cycle regulation**. Specifically, they demonstrate that impaired mitochondrial distribution during cell division can

disrupt cell cycle progression, highlighting the critical role of proper mitochondrial morphology and inheritance in ensuring successful cell cycle transition.

Reviewer 3 Comment 6 (after the initial submission)

6) In their redox biol manuscript (PMID: 32070881), the authors show that □hsp31□hsp32 cells have growth defects on glycerol similar to □hsp31□hsp34. However, only the last one has increased mitochondrial mass and post-diauxic-shift-like mitochondria, while □hsp31□hsp32 cells have wild-type like mitochondria. Therefore, the different phenotypes related to Hsp31 loss are not necessarily related. **Please comment in the manuscript**.

Author response

Thank you for the comment. Even though *Δ31Δ32* exhibits compromised growth phenotype, the mitochondrial morphology is not perturbed. It is possible that the growth defect associated with *Δ31Δ32* may be due to other uncharacterized functions.The Δ31Δ34, however, exhibits compromised phenotype due to increased mitochondrial function, suggesting that Hsp34 particularly sensitizes the mitochondrial functions performed by Hsp31 **Bankapalli, Kondalarao *et al.* ,2020** (PMID:**32070881)**. Besides, the Hsp34 is much more divergent in the sequence in comparison with the Hsp32 and Hsp33 paralogs. Therefore, we believe that Hsp34 has better evolved vital functions in conjunction with Hsp31 in maintaining overall cellular and organellar health.

Reviewer 3 Comment 5 (after revision)

"Where is the comment on the manuscript?"

Author response (rebuttal)

Please refer to line 496-502 in Discussion section.

Reviewer 3 Comment 6 (after the initial submission)

6) In their mitophagy assessments, it would be advisable to perform FM464 co staining with OM45-GFP to demonstrate interiorization in to the vacuole.

Author response

Thank you for the suggestion. We have stained the vacuoles with FM4-64 and the new figure has been incorporated in Fig. 3B.

Reviewer 3 Comment 5 (after revision)

"This is correct and convincing."

Reviewer 3 Comment 7 (after the initial submission)

7) It could be preferable to name WT-morphology as tubular, and □hsp31□hsp34 as PDS-like.

Author response

Thank you for the suggestion. We have now incorporated the changes as suggested in required figures.

Reviewer 3 Comment 5 (after revision)

"Nomenclature is still the same"

Author response (rebuttal)

As shown in **Fig. S18**, cells in the post-diauxic phase typically exhibit resistance to oxidative stress. Referring to the Δ*31*Δ*34* strain as being in the post-diauxic phase would therefore be misleading, as these cells display marked sensitivity to oxidative stress compared to PDS phase cells. To avoid this confusion, we have updated the nomenclature to refer to the mitochondrial morphology (in **Fig. 2**) as "PDS-like" rather than implying an actual post-diauxic state of cells, and the figures have been revised accordingly.

This change is purely terminological and does not affect the interpretation or conclusions of the study.

Reviewer 3 Comment 8 (after the initial submission)

8) The introduction could be shortened to the most relevant functions of Hsp31 and Ubp2 in context of this manuscript, given that no clinical aspects are addressed in the present study.

Author response

Thank you for the response. The introduction has now been shortened.

Reviewer 3 Comment 5 (after revision)

"The introduction has the same length as before (3,5 pages!!! Only 1 line less!!!)"

Author response (rebuttal)

While the Introduction has already been shortened in the current version, we are open to further condensation if necessary.
