# Supplementary material for: Ubp2 modulates DJ-1-mediated redox-dependent mitochondrial dynamics in Saccharomyces cerevisiae
Source: PLoS Genet. 2025 Jul 3;21(7):e1011353. doi: 10.1371/journal.pgen.1011353 (PMC12251144; doi:10.1371/journal.pgen.1011353)
Supplement: Attachment — Submitted filename: Review PGENETICS-D-24-00709.docx [file pgen.1011353.s050.docx]

This manuscript addresses the relationship between the PARK7 paralogues Hsp31/34 and the USP10 paralogue Ubp2. In a previous study, the authors nicely showed that Hsp31 and Hsp34 are required for redox balance, mitochondrial respiration, mitochondrial morphology and cell-cycle progression. Simultaneous loss of Hsp31and Hsp34 causes a reduction in GSH levels and consequently oxidative stress, increased mitochondrial biogenesis, and mitochondrial mass, G2/M cell cycle arrest, and increased apoptosis, for the most part reverted by addition of the antioxidant NAC. Here, the authors convincingly show that ablation of the deubiquitinase Ubp2 suppresses all defects associated to loss of Hsp31/34. They also observe a correlation between Ubp2/Hsp31/34 and alterations in mitochondrial morphology and in the MFN1/MFN2 paralogue Fzo1, and propose a role of mitochondrial fusion in this process. These findings are novel and potentially very relevant. Rescue experiments and mechanistic aspects of this rescue should be substantiated, in particular investigating causal effects, as detailed below.

Main points

1) The authors nicely discover a genetic interaction between Ubp2 and Hsp31/34. In this regard:

A) They should provide evidence that re-expression of Ubp2 rescues all Δ*hsp31*Δ*hsp32* phenotypes presented in this manuscript.
B) Moreover, is this rescue dependent on the catalytic activity of Ubp2?
C) If yes, how do the authors explain that *UBP2* deletion rescues Δ*hsp31*Δ*hsp32* phenotypes, given that Ubp2 is expected to have reduced activity in Δ*hsp31*Δ*hsp32* cells. This prediction is because Δ*hsp31*Δ*hsp32* cells have increased ROS levels (PMID: 37548361and 32070881) and oxidative stress inactivates the catalytic activity of Ubp2 (PMID: 25622294).
D) Along this line, repressed activity of Ubp2 is expected to cause mitochondrial fragmentation, but the Δ*hsp31*Δ*hsp32* cells have increased mitochondrial tubulation. How do the authors explain this?

2) Related to point 1), can it be that catabolic repression caused by growth in glucose is absent in Δ*hsp31*Δ*hsp32* cells? Consistently, mitochondrial morphology of the Δ*hsp31*Δ*hsp32* cells intriguingly resembles WT cells in post-diauxic shift phase (PDS). Therefore, the authors should test if PDS cells resemble Δ*hsp31*Δ*hsp32* cells in cell cycle progression, mitophagy, ROS levels or growth resistance to H2O2. If yes, are these effects in PDS cells rescued by deletion of *UBP2*?

3) Related to point 2) is the rescue of respiration, mitochondrial mass, cell cycle arrest and ROS levels observed in Δ*ubp2*Δ*hsp31*Δ*hsp32* cells rather due to effects in the TCA and ETC pathways? The authors could follow an approach similar to <https://doi.org/10.1101/2024.07.09.602707>. For example, is the rescue dependent on the carbon source? What happens upon deletion of *RTG1* or Hap4 overexpression?

4) Δ*hsp31*Δ*hsp32* cells have altered levels and stability of Fzo1. How do these chaperones affect Fzo1? The authors should test if this is caused by an increase in mitochondrial biogenesis.

Of note, in PMID: 32070881 they observe that Δ*hsp31*Δ*hsp32* cells have increased transcriptional of all 3 major mt dynamics proteins: Dnm1, Mgm1 and Fzo1, and very comparable increases in their respective protein levels. Consistently, in this same manuscript, they show that Δ*hsp31*Δ*hsp32* cells have increased mitochondrial mass and increased GSSG levels, thus altering the GSH/GSSG ratio. Reduction in GSH is an indicator of oxidative stress and they could revert increased mitochondrial mass with the antioxidant NAC.

5) They infer that *UBP2* deletion rescues the cellular defects of Δ*hsp31*Δ*hsp32* cells due to the ubiquitination, stability and steady state levels of Fzo1. However, besides presenting correlative data, they do not demonstrate any causal effect. Therefore, at this point, the many statements along the manuscript inferring causality are misleading (for example title of Fig. 4, statement in lines 345-346, 379-380, 410-411, 436-437, 476, 480-482, 485).

To investigate if rescue depends on mt dynamics, the authors should simultaneous eliminate Fzo1 and Dnm1, which allows to maintain mitochondrial tubulation and respiration. They should compare respiration, mitochondrial mass, cell cycle arrest and ROS levels of Δ*ubp2*Δ*hsp31*Δ*hsp32* cells and of Δ*fzo1*Δ*dnm1*Δ*ubp2*Δ*hsp31*Δ*hsp32* cells.

Of note: Δ*ubp2* cells have fragmented mitochondria, but do not affect mitochondrial mass, cell cycle progression, ROS levels or growth resistance to H2O2. This suggests that mitochondrial alterations per se do not cause cell cycle, ROS or mitochondrial mass defects.

Other points:

6) To my knowledge, a causal effect of mitochondrial fusion and fission in cell cycle progression has not been demonstrated in S. cerevisiae, which compromises a few statements, e.g. lines 320-321. Please comment/correct.

5) In their redox biol manuscript (PMID: 32070881), the authors show that Δ*hsp31*Δ*hsp32* cells have growth defects on glycerol similar to Δ*hsp31*Δ*hsp34*. However, only the last one has increased mitochondrial mass and post-diauxic-shift-like mitochondria, while Δ*hsp31*Δ*hsp32* cells have wild-type like mitochondria. Therefore, the different phenotypes related to Hsp31 loss are not necessarily related. Please comment in the manuscript.

6) In their mitophagy assessments, it would be advisable to perform FM464 co staining with OM45-GFP to demonstrate interiorization in to the vacuole.

7) It could be preferable to name WT-morphology as tubular, and Δ*hsp31*Δ*hsp34* as PDS-like.

8) The introduction could be shortened to the most relevant functions of Hsp31 and Ubp2 in context of this manuscript, given that no clinical aspects are addressed in the present study.
