# Supplementary material for: Ubp2 modulates DJ-1-mediated redox-dependent mitochondrial dynamics in Saccharomyces cerevisiae
Source: PLoS Genet. 2025 Jul 3;21(7):e1011353. doi: 10.1371/journal.pgen.1011353 (PMC12251144; doi:10.1371/journal.pgen.1011353)
Supplement: Attachment — Submitted filename: review plos genetics paper aug 2024.docx [file pgen.1011353.s051.docx]

In this manuscript, the authors show that mitochondrial defects that occur upon deletion of the DJ-1 paralogs Hsp31/34 can be supressed by ablation of the DUB Ubp2, including respiratory growth, ROS levels and mitochondrial morphology regulated via Fzo1. Consistently, deletion of *UBP2* further restores the cell cycle defects of Δ31Δ34 cells. The authors hypothesize that Ubp2 and Hsp31 paralogs cross-talk to maintain mitochondrial health under basal and oxidative stress conditions.

While genetic interaction between Ubp2 and Hsp31 paralogues is shown here, there is no functional or mechanistic evidence of how this crosstalk occurs. Further, the analysis of mitochondrial integrity and especially dynamics is rather superficial. While the findings shown here are itself interesting, the hypotheses drawn from them are not well enough explained or substantiated.

Several major points concerning the authors proposed model that could help establish some mechanistic insights:

There is no clear evidence that Hps31/34 directly regulate mitochondria. Most of the mitochondrial phenotypes could be a secondary effect from the cell cycle defect of Δ31Δ34 cell. This concern is not addressed or mentioned at all in the manuscript. Do other mutant cell lines arrested at G2/M phase also have mitochondrial defects? Can those defects also be rescued by ablation of Ubp2? How are Ubp2 levels regulated during the cycle (see also Ubp2 level in mid log and late log phases in FigS2)?

Besides cell cycle regulation how do the authors proposed that Hsp31/34 regulate mitochondrial health. Are they recruited to mitochondria upon stress? Do Ubp2 and Hsp31/34 directly interact with each other? Do Hsp31/34 interact with Fzo1? How could the known functions of Hsp31 paralogs mechanistically impact mitochondria? Are general cellular ubiquitylation levels affected in Δ31Δ34 cells?

Further points:

The manuscript should be revised, because figures are referred to wrongly, typos can be found, and citations used in the introduction do not seem to be appropriate. Additionally, some figures are lacking crucial information in their legends.

The main text mentions that the W303 yeast strain background was used while the methods only mention BY4741 yeast strains. Which background was used?

Figure 1A: is this rescue specific for Ubp2? Do other DUB deletion strains show a similar phenotype?

What is the functional difference between Hsp31 paralogues and why are specifically 31 and 34 important here?

Figure 1D. Ubp2 levels after overexpression should be shown via western blot. Does the observed rescue depend on the catalytic activity of Ubp2? How does the growth of the triple delta strain overexpressing Ubp2 compare to the Δ31Δ34 double delta cells?

Figure 2b. The quantification of mitochondrial morphology seems to be shown as absolute numbers. How many cells were counted and was this consistent between all strains? Relative numbers might be more appropriate here.

Figure 2a and b. the authors claim that 31Δ34 strains show hyperfused mitochondria. Firstly, this phenotype does not seem to be represented in the quantification. Secondly, from the pictures, this phenotype does not appear to be similar to what is classically called “hyperfused” or very interconnected (e.g. as observed in delta dnm1 cells in log phase) but rather similar to mitochondrial morphology of cells in stationary phase. The growth conditions for mitochondrial morphology are not clearly explained in the methods. Please clarify. How do other mutants with similar cell cycle defect affect mitochondrial morphology compared to this.

Figure S3. From the DIC of Δ31Δ34Δubp2 cells seem to be highly stressed and have enlarged vacuoles. Is this a consistent phenotype? Else a different picture should be chosen. Additionally, this figure would benefit from quantification.

“Interestingly, there was no difference in the total mitochondrial mass in Δ*31*Δ*34Δubp2*, and it was comparable to Δ*31*Δ*34 (Fig. 2B).”*  should refer to figure 2c. Further this is very surprising when looking at Figure 2A where Δ31Δ34 cells are clearly more “occupied” by mitochondria. NAO staining does not seem to serve as an appropriate proxy for mitochondrial mass here but would rather suggest that Δ*31*Δ*34* have a problem with cardiolipin synthesis. Please address.

Please explain clearly how JC-1 staining is a readout for “functional mitochondria”. It might be better to just refer to membrane potential and not defer any claim on functionality here.

Neither the main text nor the methods mention how ATP levels are measured. Please explain.

Figure 3 needs quantification for both panels a and b. Additionally B should include vacuolar staining.

Figure 4C: It is very surprising that all Fzo1 levels time point 0 seem to be at very similar levels in the different mutant strains. Please clarify. Is the here observed degradation of Fzo1 dependent on proteasomal activity?

4E: Fzo1 activity and stability is not merely regulated by the amount of its ubiquitylation but rather by the specific species of ubiquitin chain on Fzo1 (i.e. profusion vs degradative). These forms are not clearly visible here and therefore this blot is not appropriate to make any conclusions about the exact ubiquitylation status of Fzo1. Further, Fzo1 interacts with several proteins that are ubiquitylated themselves, so it is not 100% evident that the here shown anti ubiquitin signal is actually Fzo1 ubiquitylation.

Figure 5: What is known about the role of Ubp2 in cell cycle. Can its deletion of overexpression rescue other cell cycle defects?

Figure 6: What is the functional connection between DJ-1 paralogs and ROS. Are the elevated levels here merely a side effect of the mis-regulation of morphology?
