# Supplementary material for: Ubp2 modulates DJ-1-mediated redox-dependent mitochondrial dynamics in Saccharomyces cerevisiae
Source: PLoS Genet. 2025 Jul 3;21(7):e1011353. doi: 10.1371/journal.pgen.1011353 (PMC12251144; doi:10.1371/journal.pgen.1011353)
Supplement: Attachment — Submitted filename: answers to my review and my revised comments r.docx [file pgen.1011353.s053.docx]

**Reviewer #3: also as an attachment, called "Review PGENETICS-D-24-00709"**

This manuscript addresses the relationship between the PARK7 paralogues Hsp31/34 and the USP10 paralogue Ubp2. In a previous study, the authors nicely showed that Hsp31 and Hsp34 are required for redox balance, mitochondrial respiration, mitochondrial morphology and cell-cycle progression. Simultaneous loss of Hsp31and Hsp34 causes a reduction in GSH levels and consequently oxidative stress, increased mitochondrial biogenesis, and mitochondrial mass, G2/M cell cycle arrest, and increased apoptosis, for the most part reverted by addition of the antioxidant NAC. Here, the authors convincingly show that ablation of the deubiquitinase Ubp2 suppresses all defects associated to loss of Hsp31/34. They also observe a correlation between Ubp2/Hsp31/34 and alterations in mitochondrial morphology and in the MFN1/MFN2 paralogue Fzo1, and propose a role of mitochondrial fusion in this process. These findings are novel and potentially very relevant. **Rescue experiments** and **mechanistic aspects** of this rescue should be substantiated, in particular investigating **causal effects**, as detailed below.

Please find my comments in red and the author’s response in blue.

**Author response**

We thank the reviewer for critically analysing the current study and highlighting the novelty of the present findings.

The revised version has only very minimal improvements, because the large majority of the requests were not performed. Globally, the rescue experiments are either not presented or not convincing, and causality experiments have not been added. Moreover, the big majority of alterations stated in the point-by-point response have not been introduced, which is very annoying for a reviewer.

Main points

1) The authors nicely discover a genetic interaction between Ubp2 and Hsp31/34. In this regard:

A) They should provide evidence that re-expression of Ubp2 rescues all Δhsp31Δhsp34 phenotypes presented in this manuscript.

The phenotypes presented are:

1)Respiratory competence, assessed by glycerol growth (Fig 1),

2)mt morphology (Fig. 2A,B),

3) mt mass (Fig. 2C), functional mt mass (Fig. 2D), and ATP levels (Fig. 2E)

4) mitophagy (Fig. 3A and B)

5) Fzo1 ST levels (Fig. 4A,B), stability (Fig. 4C,D), Fzo1 ubi (Fig. 4E,F),

6) cell cycle defect (Fig. 5),

7) ROS levels (Fig. 6A, B), and mt-ROS levels (Fig. 6C,D),

8) Ox stress resistance, by spot tests upon H2O2 (Fig 7A), and Glutathione levels (Fig. 7B,C).

From these 8 phenotypes, the authors only attempt to address rescue for 4 phenotypes. Moreover, none of the 4 rescue experiments presented are convincing, as described below. Finally, overexpression is not appropriate: instead rescue experiments must be designed so that re-expression re-establishes WT-like levels. Yeast has a very big toolbox to allow it. Generally, this is achieved by centromeric plasmids. In addition, endogenous WT levels could be assessed by genomic HA tagging.

**Author response**

Thank you for the valuable suggestions. We have re-expressed Ubp2 in the triple

deletion strain (Δ*31*Δ*34*Δ*ubp2*) and analysed for the mitochondrial respiration

competency **(Fig. 1D)**.

Not answered at this stage, because Fig 1D lacks the control D31D34 EV (the controls must be on the same plate, so this cannot be replaced by Fig. 1A).

From this point on D31D34 will be named DD and D31D34Dubp2 will be named DDD

Besides, the immunoblotting and cycloheximide chase assay also suggests stabilized expression of Fzo1 upon overexpression of Ubp2 **(Fig. S6).**

Not convincing at this stage, lacks quantifications and statistics. How do the authors explain an increase in Fzo1 levels in the CHX chase, despite a rather even loading control?

Further, phenotypic analysis was performed to check for the response of cells to the treatment of H2O2 upon Ubp2 overexpression **(Fig. S7A).**

The image S7A is convincing. How many times was it reproduced?

DCFDA staining was also performed, and the microscopic analysis corroborated the observed phenotype **(Fig. S7B).**

Not convincing, due to lack of controls (as a minimum DD EV is necessary), lack of quantification and lack of statistics. Besides, please comment on the fact that DDD+Ubp2 from Sup 7B does not resemble at all the DD in Fig. 7B/C.

In fact, in Fig. 7B (and quantification in 7C) the DD has less staining for Glutathione than WT or Dubp2, and the DDD resembles WT. Therefore, in case of a rescue by re-expression of Ubp2, DDD+Ubp2 should have less staining than WT. INSTEAD, Fig. S7B shows that DDD+Ubp2 has even much higher values than the DDD EV (moreover, in S7B DDD EV has higher signal than WT EV, IN CONTRAST to Fig. 7C)

Finally, the mitochondrial network morphology was analyzed whereby Ubp2 overexpression in Δ*31*Δ*34*Δ*ubp2* reinstated the hyperfused mitochondrial structures, resembling Δ*31*Δ*34* **(Fig. S8).**

Not convincing. Lacks convincing images (I fail to see clear differences between the 4 panels) and lacks quantification.

In sum, this point 1A is NOT convincingly answered. Without the rescue the phenotypes cannot be validated.

B) Moreover, is this rescue dependent on the catalytic activity of Ubp2?

**Author response**

We thank the reviewer for the valuable suggestion. A catalytically dead mutant of

Ubp2 was constructed by mutating Cysteine to Serine mutation at the 745 residues (**Lam, Mandy H Y et al.** PMID:**19165343**), (**Santos, Clara M *et***

***al*.PMID: 39384040**). We visualized the mitochondrial network morphology upon

overexpression of Ubp2 C745S in Δ*31*Δ*34*Δ*ubp2*, and the mitochondrial structures resembled WT **(Fig. S8, panel 4).** This suggests that the catalytic cysteine is indispensable for maintaining the function of mitochondrial fusion.

Not convincing, as described above. Data for all other 7 phenotypes is lacking.

C) If yes, how do the authors explain that UBP2 deletion rescues Δhsp31Δhsp32 phenotypes, given that Ubp2 is expected to have reduced activity in Δhsp31Δhsp32 cells. This prediction is because Δhsp31Δhsp32 cells have increased ROS levels (PMID: 37548361and 32070881) and oxidative stress inactivates the catalytic activity of Ubp2 (PMID: 25622294).

**Author response**

Thank you for the response. Yes, the catalytic activity of Ubp2 is necessary for

mediating the rescue observed in Δ*31*Δ*34*Δ*ubp2*.

Although requested, this was not shown, as commented above.

As reported previously, Ubp2 is inactivated reversibly in response to H2O2 stress (0.6mM- 2.5mM) (**Silva, Gustavo M et al.**PMID: **25622294**) as an extraneous stressor. However, in the absence of DJ-1 paralogs, there is an inherent increase in the ROS levels, maybe less than what we used for the experimental condition that we genuinely believe. At this, lower ROS levels in the absence of DJ-1 paralogs plausibly do not inactivate the catalytic cysteine of Ubp2.

At this stage, this statement is a hypothesis. It should be validated experimentally.

Moreover, Ubp2 is expressed more in Δ*31*Δ*34* than WT **(Fig 1B),** leading to higher activity causing hyperfusion of mitochondrial structures without DJ-1 paralogs.

At this stage, this statement is a hypothesis. It should be validated experimentally.

Meaning: Ubp2 is ca 2X more abundant in DD than in WT. Does mild overexpression of Ubp2 (ca 2X) in WT cells resemble DD for any of the 8 phenotypes presented?

Of note: the mt morphology results presented in Fig. S5B and commented bellow, at the end of point 5, does not support the author’s statement. DD resemble PDS phase cells, and this does not appear to be the case for WT+Ubp2.

D) Along this line, repressed activity of Ubp2 is expected to cause mitochondrial fragmentation, but the Δhsp31Δhsp32 cells have increased mitochondrial tubulation. How do the authors explain this?

**Author response**

Thank you for the critical response. Ubp2 is expressed more in the absence of DJ-1 paralogs and is plausibly not inactivated by intrinsic ROS. Thus, enhanced expression of Ubp2 stabilizes Fzo1 and causes increased mitochondrial tubulation.

This assumption requires experimental validation.

2) Related to point 1), can it be that catabolic repression caused by growth in glucose is absent in Δhsp31Δhsp32 cells? Consistently, mitochondrial morphology of the Δhsp31Δhsp32 cells intriguingly resembles WT cells in post-diauxic shift phase (PDS). Therefore, the authors should test if PDS cells resemble Δhsp31Δhsp32 cells in cell cycle progression, mitophagy, ROS levels or growth resistance to H2O2. If yes, are these effects in PDS cells rescued by deletion of UBP2?

**Author response**

Thank you for your critical response. The cells in post-diauxic shift were

phenotypically assessed in the presence of H2O2. However, the PDS cells exhibit

resistance to oxidative stress **(Fig. S9)**. Cell cycle progression and mitophagy

assessment could not be performed due to the technical drawbacks in designing the experiments. For instance, in the current study, the cells were arrested in the G1 phase to synchronize the culture. However, synchronization of cells in PDS could not be performed as PDS cells were not responsive to the alpha factor.

Similarly, for mitophagy induction, cells in the mid-log phase are transferred

from dextrose-containing media to glycerol, and samples are collected over the

indicated time points. Hence, cells in post-diauxic shift could not be analyzed for

mitophagy. From the experiment performed, even though Δ*31*Δ*34* resembles the

mitochondrial morphology of PDS cells, the PDS cells are oxidative stress resistant. Previous reports also indicate stress resistance to hydroperoxides in cells undergoing post-diauxic shift (**Maris, A F et al.** PMID: **11409175).**

The author’s answer is satisfactory.

3) Related to point 2) is the rescue of respiration, mitochondrial mass, cell cycle arrest and ROS levels observed in Δubp2Δhsp31Δhsp32 cells rather due to effects in the TCA and ETC pathways? The authors could follow an approach similar to https://doi.org/10.1101/2024.07.09.602707. For example, is the rescue dependent on the carbon source? What happens upon deletion of RTG1 or Hap4 overexpression?

**Author response**

Thank you for your valuable input. We over-expressed Hap4 in the background of

Δ*31*Δ*34*and Δ*31*Δ*34*Δ*ubp2* to phenotypically assess the growth of the strains in

different carbon sources as suggested **(Fig. S10)**. However, Hap4 over-expression

showed a slight improvement in the growth of Δ*31*Δ*34*Δ*ubp2 in both glycerol and*

*dextrose*, highlighting the minor effect of TCA/ETC pathways in regulating

mitochondrial dynamics. However, more detailed studies are needed in this direction which, we believe that it is beyond the scope of the current manuscript.

The author’s answer is satisfactory.

4) Δhsp31Δhsp32 cells have altered levels and stability of Fzo1. How do these chaperones affect Fzo1? The authors should test if this is caused by an increase in mitochondrial biogenesis.

Of note, in PMID: 32070881 they observe that Δhsp31Δhsp32 cells have increased transcriptional of all 3 major mt dynamics proteins: Dnm1, Mgm1 and Fzo1, and very comparable increases in their respective protein levels. Consistently, in this same manuscript, they show that Δhsp31Δhsp32 cells have increased mitochondrial mass and increased GSSG levels, thus altering the GSH/GSSG ratio. Reduction in GSH is an indicator of oxidative stress and they could revert increased mitochondrial mass with the antioxidant NAC.

**Author response**

We thank the reviewer for the suggestion. We thank the reviewer for the critical

assessment. Indeed, hyperfusion caused due to stabilized Fzo1 also causes increased biogenesis as indicated by increment in expression of mitochondrial proteins (Tom70,Tim44, Tim23) as mentioned in **Bankapalli, Kondalarao *et a*l. ,2020**

(PMID:**32070881**). The GSH and GSSG levels have been represented as ratios in the mitochondrial and cytoplasmic extracts. Kindly refer to **Fig. 7D and Fig. 7E** in the revised manuscript.

The request in this point was to revert the increase in mt mass they observe in DD (e.g. by adding NAC to DD, as in PMID: 32070881) and then check Fzo1 levels and mt morphology.

Regarding the answer given by the authors, once again it should be noted that a correlation (simultaneous observation of increased Fzo1 and increased Tom and Tim components) does not mean causality.

5) They infer that UBP2 deletion rescues the cellular defects of Δhsp31Δhsp32 cells due to the ubiquitination, stability and steady state levels of Fzo1. However, besides presenting correlative data, they do not demonstrate any causal effect. Therefore, at this point, the many statements along the manuscript inferring causality are misleading (for example title of Fig. 4, statement in lines 345-346, 379-380, 410-411, 436-437, 476, 480-482, 485).

**Author response**

We thank the reviewer for the valuable suggestions. We have rephrased the

statements and incorporated necessary the changes in the revised manuscript.

Can it be that the authors did not submit the correct manuscript text? Because except in one case, no alterations were made.

The only statement altered, in lines 363-366 (previously lines 345-346) “Taken together, the results indicate that remission of mitochondrial structures upon deletion of Ubp2 in the absence of Hsp31 paralogs enables the cells to undergo unimpeded progression through the cell cycle.”: IS STILL MISLEADING. Replacement of “mt dynamics” by “mt structure” doesn’t solve the lack of causality.

All other statements were not altered. Therefore, as before, statements in lines 409-410 (previously lines 379-380), in lines 445-446 (previously 410-411), in lines 480-481 (previously 436-437), in line 525 (previously 476), in lines 529-532 (previously 480-482), and in lines 534-535 (previously 485) ARE NOT CORRECT, because CAUSALITY WAS NOT DEMONSTRATED FOR ANY OF THESE STATEMENTS. The manuscript only presents correlations.

To investigate if rescue depends on mt dynamics, the authors should simultaneous eliminate Fzo1 and Dnm1, which allows to maintain mitochondrial tubulation and respiration. They should compare respiration, mitochondrial mass, cell cycle arrest and ROS levels of Δubp2Δhsp31Δhsp32 cells and of Δfzo1Δdnm1Δubp2Δhsp31Δhsp32 cells.

Of note: Δubp2 cells have fragmented mitochondria, but do not affect mitochondrial mass, cell cycle progression, ROS levels or growth resistance to H2O2. This suggests that mitochondrial alterations per se do not cause cell cycle, ROS or mitochondrial mass defects.

**Author response**

Thank you for the valuable suggestion. We attempted constructing the strains as

mentioned above but could not generate viable colonies ofΔ*31*Δ*34*Δ*ubp2*Δ*fzo1*Δ*dnm1,* possibly due to the deletion of 5 genes together.

However, the Fzo1 and Dnm1 levels were measured by Western blotting as indicated in **Fig. 4A** and **Fig. S12** respectively.

This is a critical point for causality demonstration. Dnm1 dominant negative forms are available and could perhaps be integrated. Besides, S12 has no quantifications.

Further, to substantiate the role of Ubp2 in the stabilization of the hyperfused mitochondrial structure, we overexpressed Ubp2 in WT background and performed phenotypic assessment, microscopy and immunoblotting to probe for Fzo1 levels (**Fig. S5**). The results further highlight the role of mitochondrial dynamics maintained by Ubp2.

Differences are not convincing, and no quantification is presented. Besides, DD cells resemble PDS phase morphology, which is not the case in WT+Ubp2.

Indeed, fragmentation of mitochondria in the absence of Ubp2 does not cause cell cycle, ROS, or mitochondrial mass defects in the conditions used in the present study.

As mentioned before, this suggests that mitochondrial alterations per se do not cause cell cycle, ROS or mitochondrial mass defects.

Other points:

6) To my knowledge, a causal effect of mitochondrial fusion and fission in cell cycle progression has not been demonstrated in S. cerevisiae, which compromises a few statements, e.g. lines 320-321. Please comment/correct.

**Author response**

Thank you for the suggestion. We have incorporated references indicating the role of

mitochondrial dynamics in cell cycle progression in the revised version of manuscript.

Where exactly?

5) In their redox biol manuscript (PMID: 32070881), the authors show that Δhsp31Δhsp32 cells have growth defects on glycerol similar to Δhsp31Δhsp34. However, only the last one has increased mitochondrial mass and post-diauxic-shift-like mitochondria, while Δhsp31Δhsp32 cells have wild-type like mitochondria. Therefore, the different phenotypes related to Hsp31 loss are not necessarily related. **Please comment in the manuscript**.

**Author response**

Thank you for the comment. Even though Δ*31*Δ*32* exhibits compromised growth

phenotype, the mitochondrial morphology is not perturbed. It is possible that the

growth phenotype associated with 32 paralogs may be due to other uncharacterized

functions.

The Δ31Δ34, however, exhibits compromised phenotype due to increased

mitochondrial function, suggesting that Hsp34 particularly sensitizes the

mitochondrial functions performed by Hsp31 **Bankapalli, Kondalarao *et a*l. ,2020**

(PMID:**32070881)**. Besides, the Hsp34 is much more divergent in the sequence in

comparison with the Hsp32 and Hsp33 paralogs. Therefore, we believe that Hsp34

has better evolved vital functions in conjunction with Hsp31 in maintaining overall

cellular and organellar health.

Where is the comment on the manuscript?

6) In their mitophagy assessments, it would be advisable to perform FM464 co staining with OM45-GFP to demonstrate interiorization in to the vacuole.

**Author response**

Thank you for the suggestion. We have stained the vacuoles with FM4-64 and the

new figure has been incorporated in Fig. 3B.

This is correct and convincing

7) It could be preferable to name WT-morphology as tubular, and Δhsp31Δhsp34 as PDS-like.

**Author response**

Thank you for the suggestion. We have now incorporated the changes as suggested

Nomenclature is still the same

8) The introduction could be shortened to the most relevant functions of Hsp31 and Ubp2 in context of this manuscript, given that no clinical aspects are addressed in the present study.

**Author response**

Thank you for the suggestion. We have stained the vacuoles with FM4-64 and the

new figure has been incorporated in Fig. 3B.

**Author response**

Thank you for the response. The introduction has now been shortened.

The introduction has the same length as before (3,5 pages!!! Only 1 line less!!!)
